# Supplementary figures and images for: Next-generation yeast-two-hybrid analysis with Y2H-SCORES identifies novel interactors of the MLA immune receptor
Source: PLoS Comput Biol. 2021 Apr 2;17(4):e1008890. doi: 10.1371/journal.pcbi.1008890 (PMC8046355; doi:10.1371/journal.pcbi.1008890)

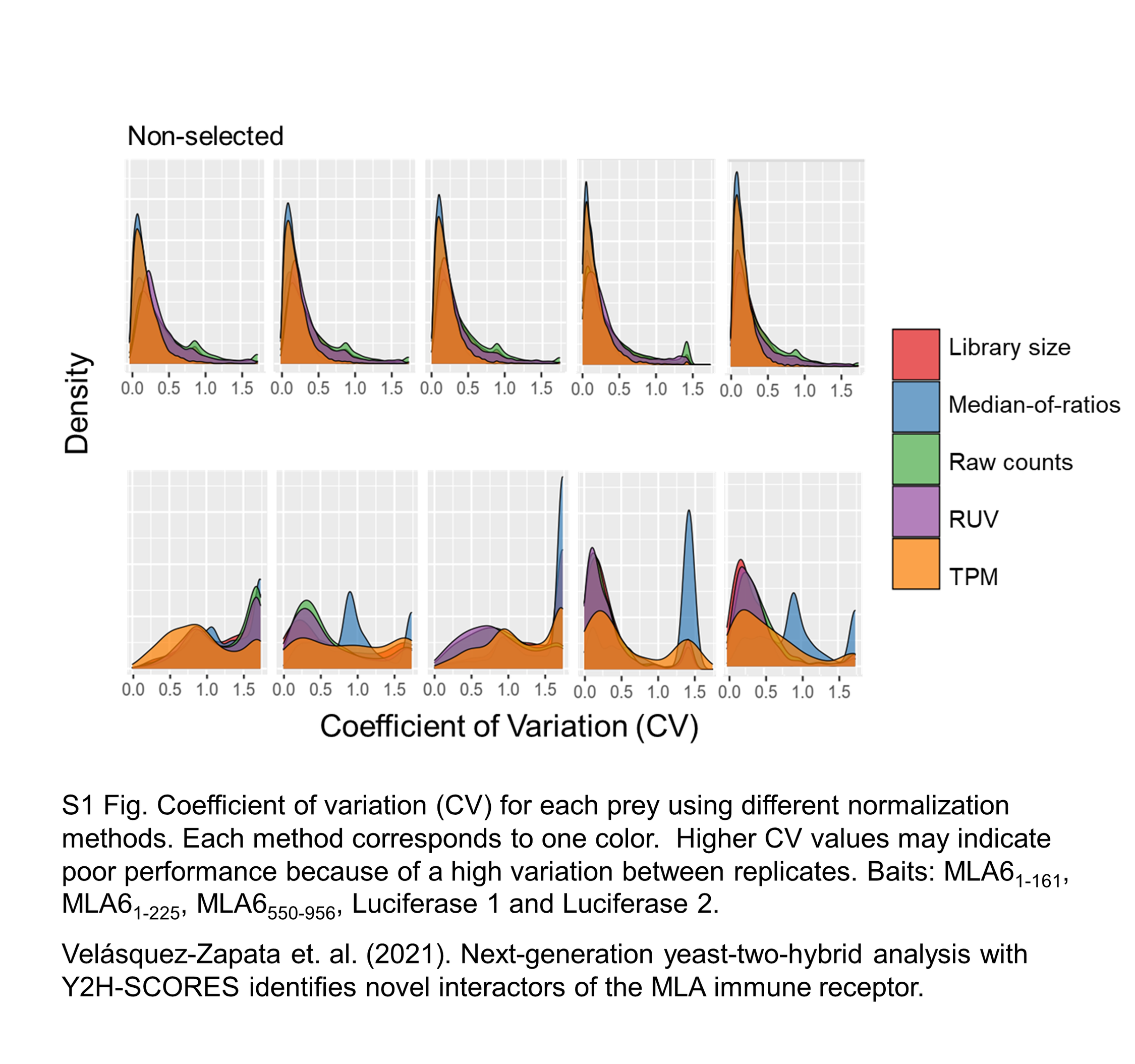

Supplement: S1 Fig — Each method corresponds to one color. Higher CV values may indicate poor performance because of a high variation between replicates. Baits: MLA61-161, MLA61-225, MLA6550-956, Luciferase 1 and Luciferase 2. (TIF) [file pcbi.1008890.s001.tif]

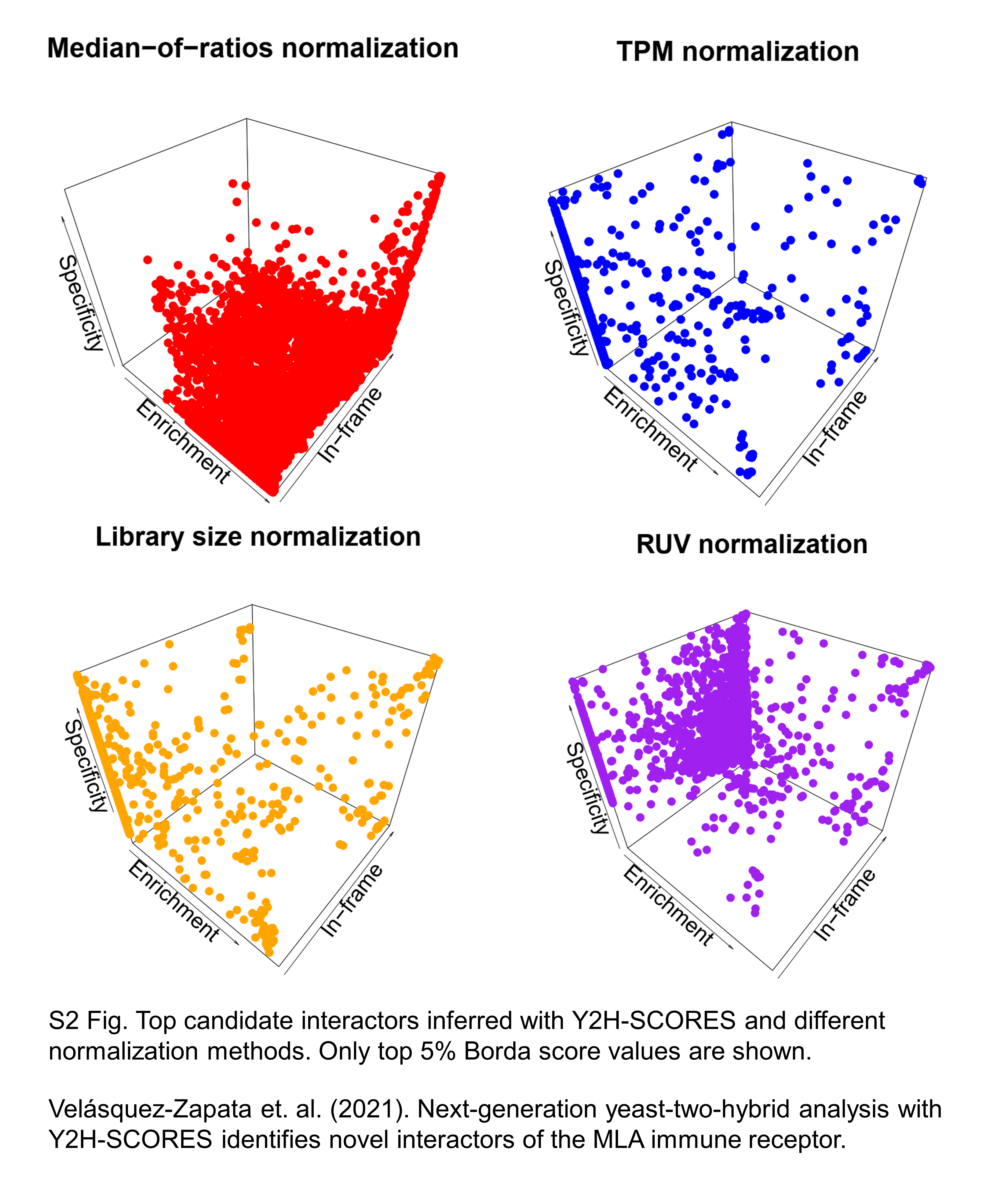

Supplement: S2 Fig — Only top 5% Borda score values are shown. (TIF) [file pcbi.1008890.s002.tif]

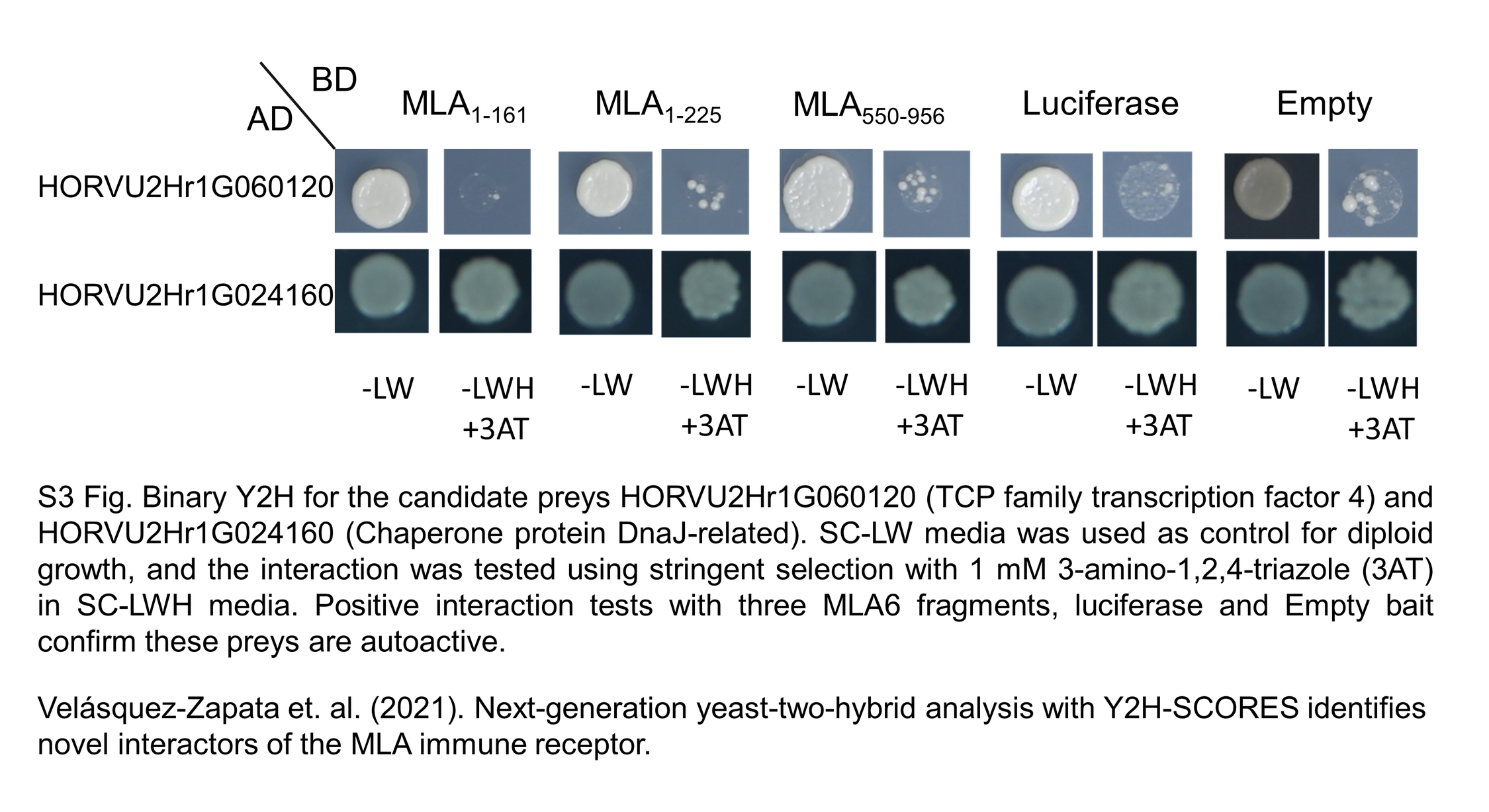

Supplement: S3 Fig — SC-LW media was used as control for diploid growth, and the interaction was tested using stringent selection with 1 mM 3-amino-1,2,4-triazole (3AT) in SC-LWH media. Positive interaction tests with three MLA6 fragments, luciferase and Empty bait confirm these preys are autoactive. (TIF) [file pcbi.1008890.s003.tif]

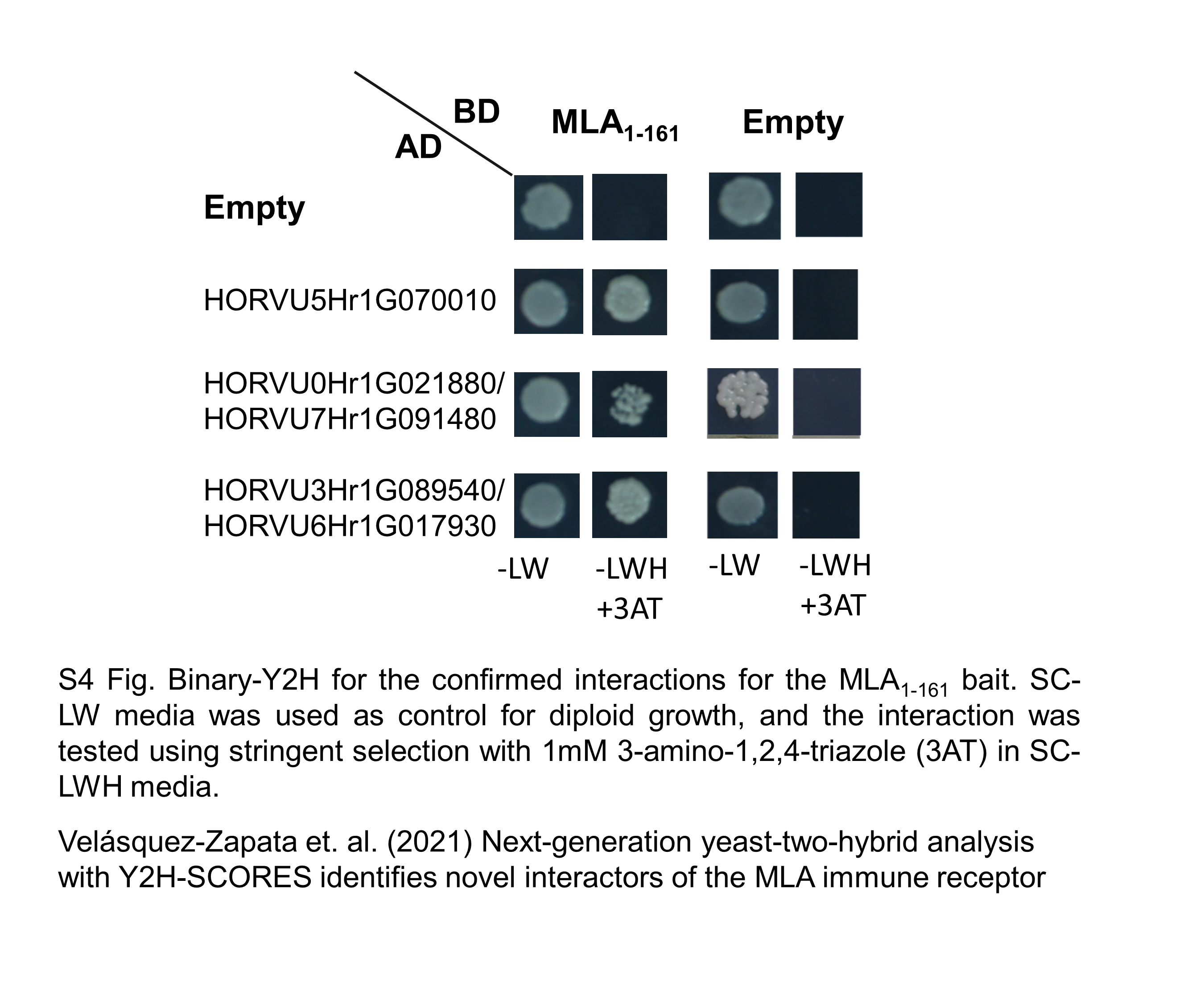

Supplement: S4 Fig — SC-LW media was used as control for diploid growth, and the interaction was tested using stringent selection with 1mM 3-amino-1,2,4-triazole (3AT) in SC-LWH media. (TIF) [file pcbi.1008890.s004.tif]

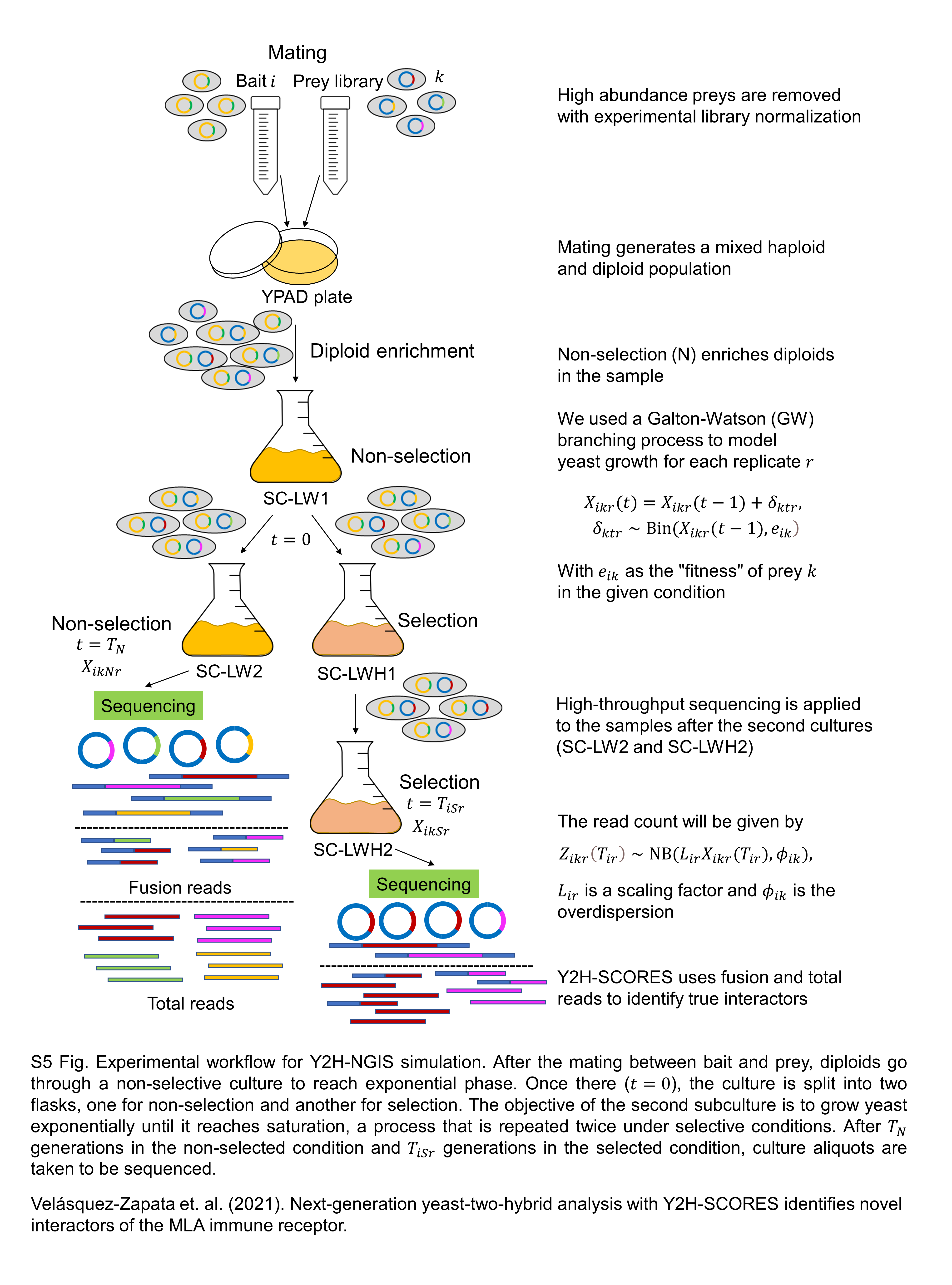

Supplement: S5 Fig — After the mating between bait and prey, diploids go through a non-selective culture to reach exponential phase. Once there (t = 0), the culture is split into two flasks, one for non-selection and another for selection. The objective of the second subculture is to grow yeast exponentially until it reaches saturation, a process that is repeated twice under selective conditions. After TN generations in the non-selected condition and TiSr generations in the selected condition, culture aliquots are taken to be sequenced. (TIF) [file pcbi.1008890.s005.tif]

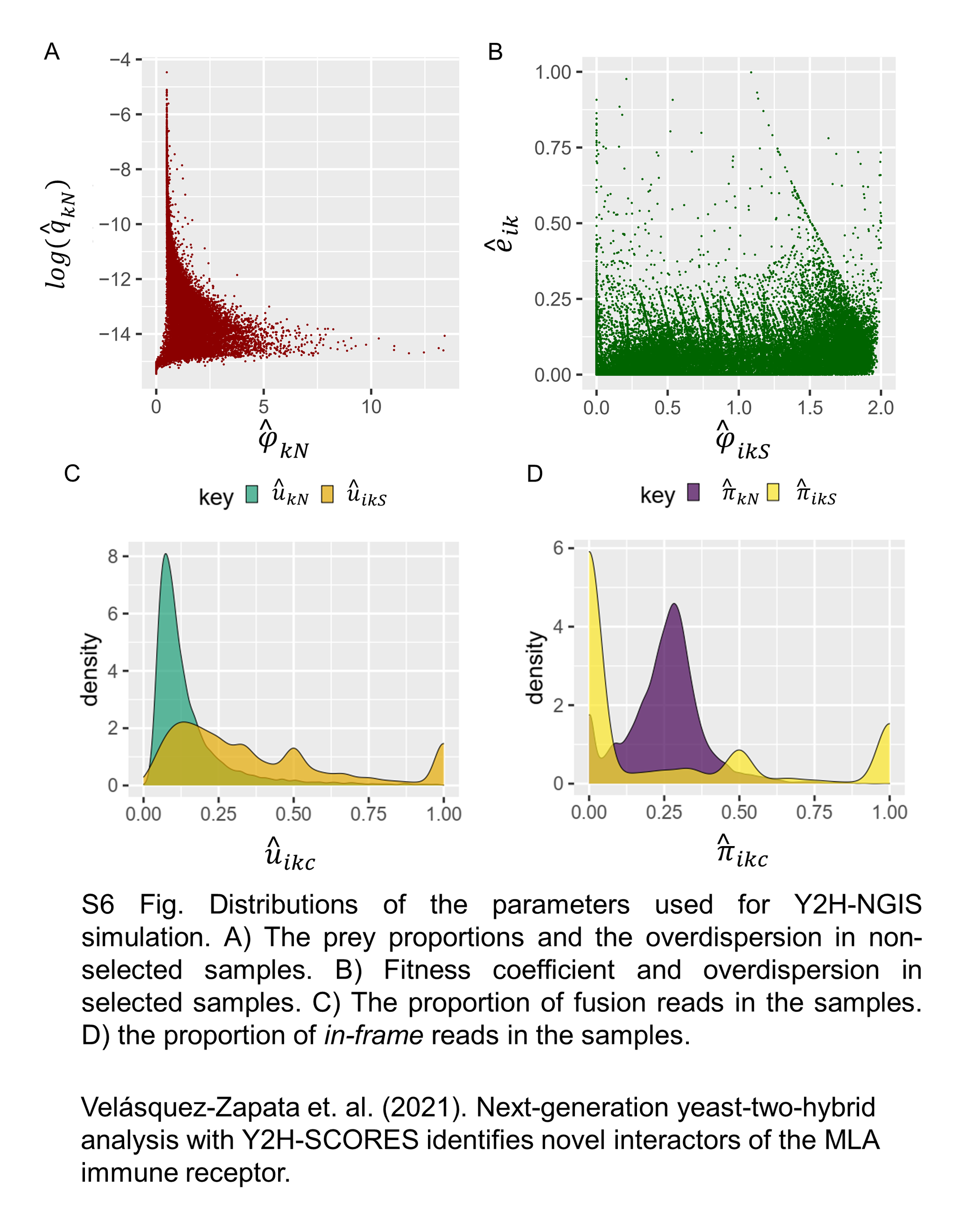

Supplement: S6 Fig — A) The prey proportions and the overdispersion in non-selected samples. B) Fitness coefficient and overdispersion in selected samples. C) The proportion of fusion reads in the samples. D) the proportion of in-frame reads in the samples. (TIF) [file pcbi.1008890.s006.tif]
