# Supplementary material for: Next-generation yeast-two-hybrid analysis with Y2H-SCORES identifies novel interactors of the MLA immune receptor
Source: PLoS Comput Biol. 2021 Apr 2;17(4):e1008890. doi: 10.1371/journal.pcbi.1008890 (PMC8046355; doi:10.1371/journal.pcbi.1008890)
Supplement: S1 Text — (PDF) [file pcbi.1008890.s017.pdf]

Next-generation yeast-two-hybrid analysis with Y2H-SCORES identifies novel interactors of the MLA immune receptor

Valeria Velásquez-Zapata, J. Mitch Elmore, Sagnik Banerjee, Karin S. Dorman, Roger P. Wise

S1 Text. $MLA6_{1-225}$ validated interactor sequences.

Kinesin-related protein 11 (HORVU1Hr1G047110)

GTTAAGATGCTTGCTGGGGAGATTGCGTTTGGTACCAGTTCACTGAAAAGACTAATTGAGC
AATCCATAGAAGACCCTGAAGGGACAAAAAATCAAATAGAGAATTTAGAGCATGAAATCCA
GCAAAAGAGGAGGCATCTGCGAGCCCTGGAACAAAAAATCATGGAAAGTGGTGAGGCATC
AGTTGCTAATGCTTCTATGGTGGATATGCAGCAGACTATTACAAAGTTGACAGCTCAATGCA
ATGAGAGGGCTTTTGACTTAGAGTTAAAATCAGCAGATAATCGTGTCCTTCAGGAGCAGCT
ACAACAGAAGAGTATGGAAATCAATGATTTACAAGAAAAAGTTCTGCGCCTTGAGGCACAG
GTCATACCAAAAACCAACATATCACCTGAACAATGCACACAGCAGGAGATTCTTGATTTGAA
AGCTAAACTTCAGTCCAAGGAAGTTGAAAGTGAGAAGCATAGATACCAGCATCTGGAAATG
ATTGACGAAAATCGAGACCTAATCAATCAGAACCATAAATTAAGTGAGGAAGCTGCTTATGC
AAAAGAATTGGCATCTTCTGCAGCTGTCGAGCTTAAGAATTTGGCTGAAGAAGTTACAAAG
CTATCCATACAAAATGCAAGGCAGGCAAAGGAGTTATTAATTGCTCAGGAGATGGCACATT
CCAGGGTTCCTGTTAGAAAGGGGCGTCCAGCAGGTAGTAGGGGCAGGGATGAGGTTGGG
ACCTGGAGTCTTGATTTAGAGGATATGAAGATGGAGCTGCTGGCGCGAAAAAAGAGGGAA
GCTGCTCTAGAAGCAGCTTTGGTAGAGAAGGAGCTTCTTGAAGAGGAGTACAAGAAGAAG
TTTGATGAAGCAAAGAAAAAAGAGCTTTCTCTAGAAAATGATCTAGCAGGCATGTGGGTTCT
TGTTGCTAAGTTGAAGAGAGGGGCTTTCAGCATATCTGATTTAAATGTTGATGACCGGTCTA
TCAATCTAACTGACATAACTAATGATATAAAGGAAAACAAAGGTGATACAAATGTAGCTCTA
GTTGAGAAACAAGTGCCAGATGATACTTTGAAGTCATTGACCGCAGAAGAATGTAGAAGTC
CAGAATTTGAACCTCTTCTTGTTCGTCTCAAGGCTAAAATTCAGGAGATGAAGGAGAAGGA
AACTGATCCCCTGAGTGATAAAGATGTCAATTCCCATGTCTGCAAAGTATGTTTTGAATCCG
CAACTGCTGCAGTTTTGCTTCCTTGTCGGCATTTTTGCTTGTGCAAGCCTTGCGCACTTGC
CTGTTCGGAATGCCCTCTGTGCCGCACACGAATCGTAGACAGGATAATTACCTTCACGTAG
ATCACTTGTTAGCCTAAGGAAACTTTTGACGCACCCAATCTCTCCCTGTAAAATCACCAACC
GTATATGTTCCTTTTTCTCTGTAGCAAAGAAGGTTACTTTTATGTTCCTTCTATGTTGTATTG
CCATTCGTTTGATTTCCCTATATAGCTTTACCTTGGCTCCTTGTTGTATTGAGAAGTTCAGC
GACTTCTCAACAAATGTGTATAGATATGTGACTCAACATTGTCCTCAGAAGTAAAGCAAATT
GATGTGGCAATACTTTTTTTTTCTTCTCTTTCAGAAGTAAAGGATGAACATGAGATCCTGAA
ACTGATGCAGCGAAAAAAAAAAAA

POLAR-like domain protein (HORVU7Hr1G113890)

GGGATTTCAAAGGCCAGCACGCCATGGCGACGGCCAACGCCGGCGGCGAAGACGACGTG
CCGGGAGACCGCAACCCCGATTCCGCCGCGTCGAGCTGCCTCACGGACGGAACGAGCC
GCGGGCCGCGACCGGCCGGTGTGAGGACCATCGTCGTCGTCATCATCATCAGGTCGCCG
AGATGGAGGCGGCGAGCCGCAGGAGGATGGACGTGCTGGAGGAGGAGTTCCACGCCGA
GCTCGAGCGCGTTCGCGCCGGCTACGGCCAGGACACGCCGCCGTTCTCGATTGGAGAGG
AG

Dynamin-related protein 3A (HORVU3Hr1G094260)

CCAAGTTTCATCGGTGGCAGCAAGGCTGTAGAGCAAGCACAGCAACAAGTTAGAGCAGCT
AGATTGCCTGCAACAGTGGTTAGAAGGGATGGAGTAGATGCAGATAGGCCACAGGCTTCT
GAAAAAACCCAAAAAGCACGTGCATTATTGGGTAGAACTACTGGTGTAAATGGAGTTATCA
CCGACCAGATCCAGCAGGGGGTACGATCGGCTGCTGAGGCAGAGAGACCAGGATCTTCA
GGTAGTGGAAGCACTTCGTTTTGGGGCTCAATATTCACCTCAAGCGAAGACCGTGCACCTT
CTTCAGCAAGAGGTAGCTCAACGAACAAATCTTATGCTTCAGCTACTCCCAACCTGGAACA
TTCATTCTCTTCAATACAGTTAAAAGAGCCACCGCTAGTCCTCAAGCCTTCAGAAAATCATT
CTGAGCAGGAGGATCTTGAAATAGCAATCACAAAATTGCTGCTGCAGTCATATTACAATATA
GTAAGGAAAAATGTCGAGGATTTTGTACCAAAAGCAATTATGCATTTTCTGGTTAATCACAC
GAAAAGGGAGCTGCATAATTTCCTCATAACTACCCTTTATAGAGAGGAGCTCTTTGGGGAC
ATTCTCAGAGAACCTGATGAAATAACTACAAAGAGGAGGCAGATACGTGATACCCTTAAGG
TCCTTCAGCAAGCCTACAAAACTTTGGACGAGATACCGCTTGAAGCGGAGACAGTGGAGA
GAGGCTATTCCCTGGATTCTGATGCGACGGGTCTACCACGTGTCCATGGGGTTTACGATG
GAAGTTCACCATATTCGACCCCGAAGCAAACAAGGCCTAGGAAATCAAGCCACTCCGGGG
AGCAGCAACAACCTTTCAGTGGCAATGGATTCTGATATTACCTTTCACGATCATCCCCTGG
ATTAGTAGTGATTTGAGCCAGCGGATGTGCTGGTAGCAACACATGATTCCGCCTTGGGTTT
GTCAGTTCGGTACCCAATAGATATATGTTTTTTTTCTCTAGCCATTGCTATTCTTTTTAGTGT
TGCGCCCAACGAAAGCATTGGCGTGGTGCCAAATTTTTGCCATGTTGCCCTCCTGTACAGT
ATATCACTGCCCAATCATTTCGACTTAAAATGAAACCGGTGATGGGTATAGTTTCCTTTTTT
GTTAAAAACCCAGGAAGAAGCCATGGGAACCTTTAGTTTTAGTGCCGAGTACTTGTATCTG
AACACGGTTAGTTTTGCCGTGATAAGTATGACATGGTTCGTGTAAAAAAAAA

Protein disulfide isomerase-like 2-3 (HORVU5Hr1G067570)

ACACACGCGTTCGTCTCGTCTCTCGCGAGCAAACTTAAATCCGCACCACCACGCCCCGGT
GACGCAGCCGATCGATCGCCACCATCCAGTCCCCCCTCCGGCTTCCAGAAGCTTCTCGAC
GACGCCATGCGTCCGGCCATCCTCGCCGCCCTCCTCCTTCTCCTCGCCGCCGCGGCCTC
CCCGGCGGCCGCGCTCTACTCCGCCGGCTCCCCGGTCCTCCAGCTCAACCCCAACAACT
TCAAGAAGGTGTTGAACGCGAACGGGGTGGTGCTGGTGGAGTTCTTCGCGCCGTGGTGC
GGGCACTGCAAGCAGCTGACGCCGATTTGGGAGAAGGCCGCCGGCGTCCTCAAGGGCGT
CGCCACAGTCGCCGCGCTCGACGCCGACGCGCACAAGGAGCTTGCGCAGCAATATGGAA
TACGGGGATTCCCAACCATAAAAGTATTTCTCCCTGGCAAGCCGCCAGTGGATTATGAAGG

AGCAAGAGATGTAAAGCCCATTGTAAATTTTGCTCTGTCGCAGGTCAAAGGTCTTCTCAGA
GATAGGTTGGATGGTAAGGCATCAGGAGGTTCAAGTAGCAAAACCTCTGGTGGCTCAAGT
GAGAAGAAAAACGAACCAAATGAATCAGTAGAATTGAATTCGAGTAACTTTGACGAACTCG
TTATCAAAAGCAAGGATCTTTGGATTGTCGAGTTCTTTGCACCATGGTGTGGGCACTGCAA
GAAATTGGCTCCTGAATGGAAAAGGGCTGCAAAGAACTTGAAGGGTCAAGTGAAACTAGG
CCATGTTGATTGTGACTCTGATAAGTCCTTGATGAGCAAGTACAAGGTAGAAGGTTTTCCAA
CTATTTTGGTATTTGGTGCCGATAAGGACAGCCCGTTCCCCTACCAGGGGGCTAGAGCTG
CTTCTGCTATCGAGTCCTTCGCGTTGGAGCAGCTGGAAGCGAACGCTGCACCACCTGAAG
TTTCTGAGTTGACTAGCGCAGATGTGATGGAAGAGAAGTGTGCTTCTGCTGCCATTTGCTT
TGTGTCTTTCCTTCCAGACATCCTGGACTCAAAGGCAGAAGGAAGAAACAAGTACCTTGAG
CTGCTACTATCTGTTGCTGAGAAATTTAAGAAGAGTCCATACAGTTTTGTCTGGGCAGGCG
CCGGCAAGCAAGCTGATCTGGAGAAGCAGGTCGGAGTCGGTGGCTATGGCTATCCAGCC
ATGGTCGCTCTCAACGTGAAGAAAGGCGCATACGCTCCGCTCCGTAGCGCCTTCGAGCTC
GCCGAAATCACCGAGTTTGTGAAGGAGGCAGGGCGCGGCGGAAAGGGCAACCTTCCTCT
GGAGGGCGCCCCGACGGTGGTCCAGTCGGAACCATGGGACGGCAAGGATGGAGAGGTC
ATCGAGGAGGACGAGTTCTCGCTCGAGGAGCTCATGGCGGACAGCTCTGCGCCCAACGA
CGAGTTGTGATTCTCGAACCATGGTAGCGTTTAGAGGAACACTGATGCTTCACTTGTTCTG
ATGTGTACACTGTACAATTTGGACGGGACACTTTTTGGAATGCCTTTTCTTTATCTCAATGTT
AGCATTGTGGGAGAAAGCTCTGGTGTGCAAGGTGTGGGGGTGTCCTTGCCCCCGAAATAA
AACCAGTTCTGAGCTTAAAAAA

Basic helix-loop-helix (bHLH) DNA-binding superfamily protein (HORVU1Hr1G071330)

CCAGCGGATGACGCACATCGCCGTCGAGCGCAACCGGCGGCGACAGATGAACGATTACC
TCAGGGTCCTCAGGTCCCTCATGCCGGGATCCTACGTCCAGAGGGGAGACCAAGCATCAA
TCATAGGCGGAGCCATAGAGTTCATACGTGAGCTAGAGCAGCT

WPP domain-associated protein (HORVU3Hr1G032920)

GCACCGCGGCAGGAAAGTGGCGTGCCTCCAAATTCCAACCTAGCAAACATGGCAGAGATC
TTGGATGTGCCAATCAGTGGCACCAATGGAAGTGCCACTGTTCAAGCTGAACAGAGCTTGA
GTGTGCCACTCAGGGACACCAACGGAAGTGCCAGTGTTCACGGTGAAGAGAGCTTGGATG
TGCCAGTAAGTGATACCAATGGAAGCGCCACTGTTCAGGATGAAACTTCTTCGGAAGGAAA
TGAGTTAATCATCGTGGATGAGATGGACTCCTTGTGGGATGATGTAAATACCATGGTTGAT
ATCTCAACATTCGTGACGTACTCTGTCATCAAAGGGTTTGTAAAAGATGCCGAACAAGAAAT
AGGTCAGCAGCTTGCTTCCAAGGATGAAGAGATAAGGTTGCTGAATCAAAAGCTGATGCAG
CTTGGAAATGGCAGCCTAAGTTTGTCTGGGGGTCGGGATAGAAAATATGATGAAGTTTATA
GTATCCGCCAACAGCTTCATGCCATTTCCAAGTCACTCTTGAATTCTGAGTGGGGTCTTTC
GGGGTCCCAGTATAACTTTGATGGTGCAGATGATGTGAGTAAACTTAGAGACAATGAACAT
TCCTCCAGAAATGGTTCTGCAAAGGTTGAAAGTGCTGGAGCCTCTCCTGATGCGGCTTTCG
CTGATGCGTCATGTTTGAAGCACTTGGATAGGGATGCTTTGATAGCCCATTTTAATAAAGAG
ATGAATACTATGAAAAGGATGCATGATAAAGTCGTGGAAATGAAGACAGAAGAGATATTTG
CACTCAAGCGAAACCTGCTAAACAAAGAAGGATCCAACCCATGGCATTTACGGAATAACAA

AGAATTCGAGCAGATTAGAAAGAAAATTGGGGAAGTTATGACAAGATTGGATGGCCTTCTC
ATGGAGAATAATAAGAGAACCACTTCTGGCGTCAAGGCAGAAACATTTGCTGGCCAACAAG
ACAAGAAAAATGTTTTAGATTCTGAAATCCAGCAAATACAAGGTGCTGCCACTAATAATCAA
GTAGAAGTGTGTGCTTTTCCTACACAGGCATCACACATTGCATCCATAGAGGCAGATCATG
CAAAGAAAATTGGAATGTTAGAGTCTGATATTGAAGAAGCCAGGATGGCAACCATGATTAG
AGAAGAGATAGAGATGATTGTACTAAGAGAGTTCGTTAACGAAATAGAAATACGGTTGCAT
GGTAATGAGATGGAGCATAACATGAAGCAAGACATTTGCTCAGTTATTCAGAATGAAGCTG
TAGCAGAAGCAGTGTTAAACCTCAACTCTACATTGTTGAAGTACAATGAGGAAAAGAGCTG
CTCTGAAGCGGCATCGACTATACAAAAGCAGGAGATTGAGAATCTGAAGCGAGCTGTTGA
CTCTTTCAGTAAAGTAGTGAGAGAAAAAGAGGAGTGTCAGATTGAATTGGGAGCAATGAAG
GGTCATATGGATTTATTATCCCATCAACTTGATTTACTTAAAGTCAAAGTGGAAAAGCAAGA
CTCCTGTATATCTGAAAAGAACAAGGAGTTTGATATGATTGTCGGCAGAGTGGAGCAAGCT
CTGCAGCATGTACGTCAAAATGAGATCAATATGAGCGAGTTCCATGACAGATTTAGAAATG
CTACAGACTCTCTGAAGGAGGTGGAGAAACAAAATCATTATTTATGTAAAGTCATCGAAGA
GAAGGAGAAAATATTCACATCAACCATTTATAAAGAAAAGGAGTTCAAAGAACACATGACAA
GTCTTGTTGAATCCATGAGAGAGTTTGAGAATCTTGTTACAGATCAACAAGCTATCATTGCA
AACAAAGTTCAGCACAGTGAATCAAGGTTCTGTTTACTGAAAGACCAATGTAAACACCTCAT
GAAAGAAGGCAATCTTTTGAGAAGGAAGGCATTGCGATACAAGGAGATATCTGAGACAAGA
GGCTCTAATCTTCAAAAGGCTGAGCTCGAGGTGGATTTACTTGGTGATGAGGTTGAGGCCT
TAACGGATCTTCTTGCAAAAATCTATATTGCGCTCGACCACTATTCTCCAGTTTTGCAACAC
TATACTGGAGTTATGGAGACCTTGAACATGATCAAGAAACACATAAGCACAGCAAAGTAAA
ATGCCTTATGCACATTTACATCAGCTGGTGACATGTTAACAGCAGAATTTGGTCTTATTGGC
CAGAAGGTGCACTTCAGACGATCTGCGGTAACTTTTTTAGGCGGTTGATGCAGCCCTTGCT
GTAGAAACCAAGGGCTGTGTAGTGCAGAATAGCTTGTTCCCCTCCGTAGGCAGTAGATTTT
GCTAGTCTTTGAGCACCTTGCAACTGACCCACCCAAACCATGGCTAGTCCTTCCTTGTGCG
TGTCTCTGAGCTACATTCTGATGTATGGGGTTGTGTCACCTGCTGATCTGCAAAAGTGAGA
CTTGAGTGACCAGTAACAACGGTTGTGGAGTGCTTGTGACCAAATTTTCATTCGGTTTATGT
AAGAGCTTGACATCTGAATTTGATGTTGTCTCAGTAATCTACAGACTCTGCTGCTTTTTTTT
TAATCTTGATATACACGGTGGCATCTAAAAAAAAAAAAAAAAA

AAA-ATPase 1 (HORVU0Hr1G021880)

CATTGCCAGCTTGTGTGAGGGCTTTACTGGGTCAGACATCCTAGAACTGTGCAAGCAGGC
TGCATTCTATCCTATCAGAGAGATTTTGAACAGCGAGAAAGATGGGACAAGAGCAAACAGT
CCAAGAGCCTTGAGGCAATCGGACCTGGAGAAAGCTCTTTCAACATCTAGAAAGGGAAAG
AAGGCCGCAAGCGGTGCGGCGTCAGGTTTCCAGTCTCCTGTGTGGACCCGTCCGTCGGA
TCCTGAAGATGACCAGGTACAGAGCGCAATCT

Homeobox-leucine zipper protein family (HORVU4Hr1G078410)

TCGAAATTGGCATGGGCATTCGTCCTGCTCACTCTATGCTCCAACAGCATGCGCGGGGAG
AAGCTGGCGTTCGCTGAGAAAGAGAACCTGCTGGCGGCCTACATGGATCACGAGGAGCC
CGAGCTGGAGGAGGCCGACGAGGAGGAGGAGGAGGAGGAGGAGCGCGCCATGTCGTGC

GGGCTGGGCGGGAAGAAGCGGCGGCTGGCGCTGGAGCAGGTGCGCGCGCTGGAGCGC
AGCTTCGAGACGGACAACAAGCTGGACCCGGAGCGCAAGGCCCGCATCGCCCGCGACCT
CGGCCTGCACCCGCGCCAGGTCGCCGTCTGGTTCCAGAACCGCCGCGCCCGCTGGAAGA
CAAAGCAGCTCGAGCGCGACTTCAACGCCCTCCGCGCCCGCCACGACGCGCTCCGCGCC
GACTGCGACGCGCTCCGCCGCGACAAGGACGCCCTCGCCGCCGAGATACACGAGCTGAG
GGAAAAGCTGTCGACCAAGCCGGAGACGGCGGTGAAGGCGGAGGCCACCGGCAATGTC
GAGGCCGCGGAGGAGCGCCTGCAGCAGGCGACGATGGTCGGCGCGGCGGTCTGCAAGG
ACGGATCCTCGGACAGCGACTCGAGCGCGGTGTTCAACGACGAGGCGTCGCCCTACTCC
GGCGCGGTCTTCGAGCAGCAGGGATTCATGGGGTTCGGCGCGTCGTTCCTGGACACGGC
GTCGGCGGCCGCGGCCACCACGGGCTGCACGTCCCTGCCCATGCTGGAACCCAAATGGC
CCAGCGCGTACCCGTACGACGCGAGCAGGTCCAGCGCCTACGGCTTCACGGAGGAATGG
CTGTCCGGGTCGGACGCGATTGGCAACGACGGCAGCTCCGCCTTCTTCTCCGAGGAGCA
CGTCTCCAACCTCAACTTCGGCTGGTGTAGCAGTGGCGCCGAGGGTTTTGACCTCCAGAG
TTACTGTAAAAAGTAAGACGGTGTCAATGTCCATGCAGCGACAGCGGGTACTTACTTTCTA
CTGGCTAGCTACCTTAGCCTAGCTAGCGGACAAGGAGAGAGGTTAGCAATGGTGGAATTC
GATGAGAAGGGTGAATTGCCGGATGAATGGACCTCCTGCCTGCCATTGCCATGGAGGGAG
GACGTAAATGTTACTACTAGTACACAGTACACAAATAACGGTCTCTAGGTTTAATCGATGGT
GTAAAAAGTTGCAATCCAAGCATGCAGCCGTCCCGAAAAAAAAAAA

Ubiquitin system component Cue protein (HORVU0Hr1G022500)

TCTGACATGGACGATGCACGGGCCCGTGCATCAAGAGCTCTGGAAGCCTTGACGAAGTCC
ATACTGGAGGGTGCAGGAGCTGAAGCAGCGCAGAGCTTGCATCAGGAGAACATGATGCTC
AAGGAGCAGATGACGGCCGTCCTGTCGCAGAACGCGGTCCTGAAGCGCGCGGTGGCAAT
CCAGCATGAGCGACAGAAGGAGTTCGACGAGCGCAGCCACGAGGTGCAGGGCCTGAAGC
AGCTCGTCCTGCAGTACCAGGAGCAGCTGAGGACTCTTGAGATCAACAACTACGCGCTGC
AGATGCATCTGAAGCAGGCCCAGCAGAGCAGCTCCATGCCCGGGCGCTACAACCCGGAC
GTCTTCTAGTTTGGCTTCAGGCTGATGTTGATGAGCCCTGGCCCTTGGGGATTGAGAGTTG
AGACTAGTGGTACTCTGCCGTATGATGGTGAAGATGACTAGAAAATGTTGTTGCCTGGGAT
TCAGACTAGTTGGGTGGATGTTGGTTTGCTGACCACCTGTTGGCCGTGGGATGGGGCGCA
AGGCTCCCATGTAGATGATATGTTGGTTCAAACCTGTTGCGTTTAAACGTTTTCGTATTTAA
TATATACACAAAACAATCCAGATTCTTTGCAAAAAAAAAAAAA

Kinetochore protein NDC80 homolog (HORVU5Hr1G114950)

CAACTGTCGGCGCCCTCTCGCCTCAAGGCGATGGAGGAGAAGAAGGATGCATTTACTGCC
GACGTTCAGAAGTTTGAGGCGGTGGTGGAGAGCTGGTCCACCAAGATCAAGGAGAAGGAA
GAGGCTTTGCTGGAGAAGGAGAAGGAGCTTGAGGCCAAGGTGTTGAATTGCCAGCAGATC
ATGGCCGAGAATGAGGAGCTGGTGAAACAGGTGGAGGTGCAGGTGGTCAATGTGAGGGA
CGTCAATAGGATGGCTAGGGAGATGCAAGCGGTGGAAAATGATATTGCCAAGTTGGAGAA
TATGAATGCCGCACTGGAGGAGAAGGGGTGGGAACTCGAAGCTGCGCTGGTCAGTAAGC
TTGAGGAGATCGAGGGCCTTGCCGAGCTGTGCAACCAGTCCCTCAGAAAGTTGAAACCCA
GGATTGATTTCCAGTACGAGGTAAATTCAAAAGGATCCTCCCCGGCTGAGATCCTTGGTAC

CACCTACAAAACCGCCCTGAAGCCCGCACTCAATGCTCTTCTTAGTGAGACTAACATGCTA
GTTACCTCAAAGCATGGTGAGTCAGTTGATCTACAAAAGAAGTTGCAAGGAATTGTTAACAT
GTTGGAGGAGAAAAGGAATCATGTTTCTGTTCTACAGGCTAAACATAACGAGATGACTGCT
CAGATGGACTCACTGGATCGTGAAATCCAAAGCCATGTTTCACGCTGCGCAACCGATGCTA
GAAAAATGAAAGACAAGTTTGAGGAAAAAGAACATCACCTGAACACCGTCGAGAAGGAGG
CAGAGGTCTTTTTAAAGAACTCCGAGGAGGGTCTCCAAGCTGCGTTGAGGGAGACCGACG
AAGAAACCCAGATGTGCGCCAGGGAGCTGCTCAAGCTCATCGATTCCATCTCGGAGTACA
AGGAGTTTGTGGAGCTGTTGATTGCCGAGATAGTTGAAGAGCTCCACGAAAGCGCCGAAA
ACATGGCTTCGCTGTGATCCAAGACTCTGGTGTGAGGTCACAGGTTTGAAGCGAGTTGTAT
TTGGTGCTGCTGCTGTAGAGTTTGTCCGTACGCGAATAGGTACCTTATTGGATTGTTTAAC
GTCGTCTTCGTTGTAGTGTGATAGTTTGAGGGCTTGATTGGAGTTGTAGTCGTGCGCAGGT
GAGATGAGTTTGGTGTGCGTGTTTCTTGCTGCATTGGCCAGATGAACATGCAGAGTACTCC
CTTCATTCCCAAATATACAAGTCCTTTTAGAGTTTCCATTAGGGGACTACATACCGAACAAA
AGCATGTTTATATACATCCGTATATAGATTTTCAATGAAAT

OBERON-like protein (HORVU5Hr1G027950)

GGGTTTTGCAACCTTTGCATGTGTGTGATTTGCAACAAGTTTGATTTTGAGGTCAACACATG
CCGATGGGTTGGGTGCGATTTCTGTTCCCATTGGACACACACTGACTGTGCGATTCGTGTT
GGCCAGATTGGGACAGGGCAATCAATTAAGAGCAGCACCGGTCATGCGGAAATGCTTTTT
AGGTGCCAGGCTTGCCAGAAGACATCAGAATTATTCGGTTGGGTTAAGGATGTGTTTCAAC
AATGTGCTCCTGGTTGGGATAGGGATGCCTTAGTACGAGAGCTTGAGTTTGTTTGTAAGAT
ATTTCGTCTAAGCGAAGACCCAAAAGGAAGAAATTTGTTCAGGAAATGTGCGAATCTGATT
GAAAGATTGAGGAATAGTTCTCCTGATTCTGTCAATCCTAGGATGATACTGCATGCACTTCG
AGAGCTTGAGATGGATTCCCTGAAGAGCTCTGAAAATGAAGAATCAGGACGCTTGATCACT
CCCCAGGAGGCATGTAATCGTATTGCAGAGGTAGTCCAAGAAGCTGTCAGAAAGATGGAG
CTTGTTGCTGAAGAGAAAATGGGACTGTACAAAAAGGCTCGCACCGCCGTGGAGGCCTGT
GACCGTGAGCTTGATGAGAAGGCCAGACAAGTTCAGGAGTTCAAGGCCGAGAGGCTGCG
GAAGAAGCAGCAGGTGGAGGAGCTCGAAAGCATTGTTCGGTTGAAACAGGCCGAGGCCG
AGATGTTCCAGCTCAAAGCAAGCGAGGCCCGCCAGGAGGCTGAGAGGCTCCAGAGCATC
GCGCTTGCCAAATCTGAGAGGGCTGAGCAGGACTATGCTAGCCTCTACCTCAAGCGGCGC
CTGGAGGAAGCGGAGGCAGAGAAGCAGTTTCTTTTCGAGAAAATAAAGCTTCAGGACGGC
CATAGGCCCCCGCAGGCGAGCAGCAGCGTGCCTGGTGATTCCTCTCAGGCGCCCTCTCA
GGCGTTGATGCTGTCCAAAATTCAGGACCTTCTCAAGAACGTTCGTACCATGCCGACAAAG
TCGGAGGCGCATTCAAAATAAGAGCAGCAGTGCTCATCAGGTTTGGGATTACATGTTTGTG
TTATTGTGCTAATGTATGGTGACTTAAGTGTGATGTGTTAGGAGTATAATGGGCGGTGCTC
CCCCTTGGAAAATCAAATATTAATGTTGTTGGCTATATCGGGATATGATATACTCATGTAGG
CTACATATTTTTAATTGTTATTACTTTGAGTAAGAGATC

Golgin 5 (HORVU1Hr1G086390)

AGGAGAACGTCTGCAGACAGTAATATGTCATACTATCTGAGGAGCATGACTCCAAGTGCTT
TTGAATCAGCACTCCGTCAAAAGGATGGAGAATTGGCTTCCTACATGTCACGCTTGGCCTC

ACTGGAATCTATTCGGAATTCCCTGGCAGAGGAGTTGGTAAAACTGACGGAACAATGTGAA
AAGTTGCGGAGTGAAGCTGCTGCTCTGCCTGGCCTAAAGGCTGAACTGGAAGCACTGAAA
CAAAGACACTTTCAAGCTCTGGAACTTATGGGGGAACGTGATGAAGAGTTGGAAGAGTTAC
GGAATGATATTGTTGACCTAAAGGACATGTACAGAGAACAAGTGGATCTTCTTGTTAGCCA
GCTCCAGACATTGGGCGCCCGTGTATAGTGCAATTGACCCAACAAGATAACAGTTTTGAAG
GCCCGAACATGTTCCGTCAGAGTTGAAAAAAGCAAAACCCGCGGCGAGTGTGGGAAGTTG
ATAGTCATACGAGGAAGATATATCTTTATTGATTTTGTGTACATCGTGAAGGATCTTTGTTGT
CGGTCTCGGTAGCATAGCCAGTGTGTTGTACACTAGACTGGATAGCTCCACAGCTCTACAT
TCATTGATGACTTGCATGCAAAGTTTTTCGTTCACAGAAGAGAAGTGTGTCATCGTCTCAGC
TAATAAATACTACTCCCTCCGTTTCTAAAAAAA

Unknown function (HORVU5Hr1G070010)

GGCGGAGGCGTAGTCTACACGGCCGTGCCGTTCTACAAGAGAGCCAGGCAGCTCGAAGA
CAAGGCGATCGAAAACGTGGAAACTGCTTTGGACGTTTTGGAGCGTGCCTCTGAGGTGAC
GGAGAAGTTTGCTGCTAATGTTGCCAATTCCCTACCGAAGGATGGATCCCTGCACAAGTTG
GCTGAGGAGCTTGAGTACATTGCTGAGGAAGTCGATAAGGATGCACACAAGGCTGAAGTC
ATGATTAAGAAGATCGAAGCGCTCAGCGACAAGATTGACGCCGCGGTGGAGCCTGTCATT
AAAGAGCTCGAGGAGGAGTTCAAGCCAAAGCCAGCATCCCACTCTGGGTCAGACGCCCAG
AAATGACCGTTCACATAAACTGTGGGAGAACGCTCCCCAAAGTGTCCGCTGTTGTACATAT
ATGTTGCTGGTTTTTCTTTCCTTCTTTCGAAGTGCCAACTTGTAAATATGTTCGTTGTGCCCT
ATGAACTAGAAGGACCCCATGGTTACAGTAAGAAATGGTGATCGTGTGCTGGGTGCCCCG
ATCTGAAGCACCCTGTAATCCTGATAATGTTACAGTAAGAACACTGCATCTGAAGAATGGAT
ATGTATGTGCGTGCATCAACTTCCGCCCTAAAAAAAAAAAAAAAAAAAAAAAAA

Pentatricopeptide repeat 336 (HORVU6Hr1G017930)

TCGACGACGATGTGGCGTTCCCGAGCTCGCGCTCTCCTCCTACTCCGCTCATCCGTCCCC
AGGTCGCCACCGCCGCCGCAAAATCCACTTCGGAGCTTGACCCGAGCCCCGCCCCCGCC
ACCACGCCTTCTCTCCCGCTTCCTCTCCTCCTCCTCCCCGGAGGCCCTGCCGGACGCCCC
CTCCTCTTTTGCGGATGCCCTCCAGGATGATCCCTTTGCCTTGGCGCCCGGCGTTGAACC
CGGATCGGCTGATCCCACTGAGGCCGACGAGGACAACCTGGCCGCGCTCTGGGAGCAGG
ATGCGGGCGACGCCAACGACATCTTCGTCTCCCCCGCCTCCCCCGACACCGCTGACTCAG
AGGCCAGCGACCAGGAGGTCGCCCGCGTCCGCGCCGTCGTCCAGTCCACCCCGGAGGA
CCAGATCCCCTCCGCCATCGCCGACATGGTCGTTGACTTCACCGAGCCGCTCCTAGCCGC
CATCCTCCTCTCCGCCGAACTGCTCTGCAAGGTTGCTGATTCGGCCGAGATTGACAAGAT
GGACGCTTACATGCTCTGGGATTTGGTGAAGGAAATGGGCAGTGTGCCTGGATCATTGAG
CACCCCACTGTTGAACAGAGTGCTAGCAATGTTCTGGAAGCTTCAGAAATCGAAGGCGGC
GCTGGAGGTGCTTGACAAGTTCAGTGAGTTTGGCTGCACTCCGGACGGTGACAGCTATTA
TCTGGCGATTCAAGCGGCTGGAAAGAAGTCCATGGTTGGCGCCGCATGGGGAGTTTGCGA
GAAGATGATTAGCTCTGGGTGCTTCCCTGATGGGAAGAAGACTGGTGAGATTGTGACCTTC
TTTTGCCAGGGGAAGATGGTGACGGAGGCGCATTCAGTATACCTAGCAGCAAAGGAGAAG
AAGGTTCAGATTCCAACATCAGCCTTGGACTTCCTTGTTGGTGCTTTGGCGAAGAATGATG

CGACCATCACTACGGCTCTGGAGCTGCTTGGGGAATACAAAGGAGACTCTCTTAAGCATG
CGGGCAAGTCCTTTGCTGCTGTTATACATAGTTTGTGCAGGATGAAGAATGTGAATGATGC
AAAGAAGCTGTTGATGAGGATGGTGAATCTTGGCCCAGCTCCTGGTAGTGCAGTGTTCAAC
TTTGTCATCACAGGATTGTCTAAAGAGGGAGAAATGGAAGACGCAAAGGATTTGATAAGAG
TGATGGAGAGCCGAGGGCTGCGCCCTGATGTTTATACATACAGTGTGATAATGAGTGGCT
ATGCAAAGGGAGGCATGATTGATGAAGCCCATTCTCTACTTCGCGAAGCTAAGAAGATCCA
TCCAAAACCAAGCAGGGTTACTTATCACATTCTAATCCGTGGTTACTGCAAGATGGAGGAG
TTTGAGAAGGCCCTGGAGTGCCTAAAGGAGATGAAGGAGGATGGGTTGCAGCCAAACATG
GATGAGTATAACAAACTCATCCAGTCGTTATGTCTGAAGGCTATGGACTGGAGGACAGCTG
AGAAGCTCCTTGAGGAAATGGAGGGCAGTGGGTTATGCCTTAAGGGCATTACCCGCAGCC
TCATAGCAGCAGTCAAGGAGTTGGAAATGGAAGAGGCGTCAAAAGACAGCCAAGAAGCAT
AGTTTCTCTAGGATACGCTTGAAGTGACACAGCCACTAATCAGTTTTGCGGATCACCCTCTT
TTTGCTCTCTGCATCTCTGGTGAGGTTATGAAAAACACCATCTTTTGTACTTGCTGCTTGTT
GTTAGATGCTAAAATAATATTGCATTTGTATTATAAATGCTGGACCTGAACGGATGCATATTT
AATTTTGGTCTAAAAAAAAAAAAA
