# Supplementary material for: Next-generation yeast-two-hybrid analysis with Y2H-SCORES identifies novel interactors of the MLA immune receptor
Source: PLoS Comput Biol. 2021 Apr 2;17(4):e1008890. doi: 10.1371/journal.pcbi.1008890 (PMC8046355; doi:10.1371/journal.pcbi.1008890)
Supplement: S3 Text — (PDF) [file pcbi.1008890.s019.pdf]

Next-generation yeast-two-hybrid analysis with Y2H-SCORES identifies novel interactors of the MLA immune receptor

Valeria Velásquez-Zapata, J. Mitch Elmore, Sagnik Banerjee, Karin S. Dorman, Roger P. Wise

S3 Text. Y2H-NGIS experimental protocol.
Adapted from Pashkova et al., (2016).

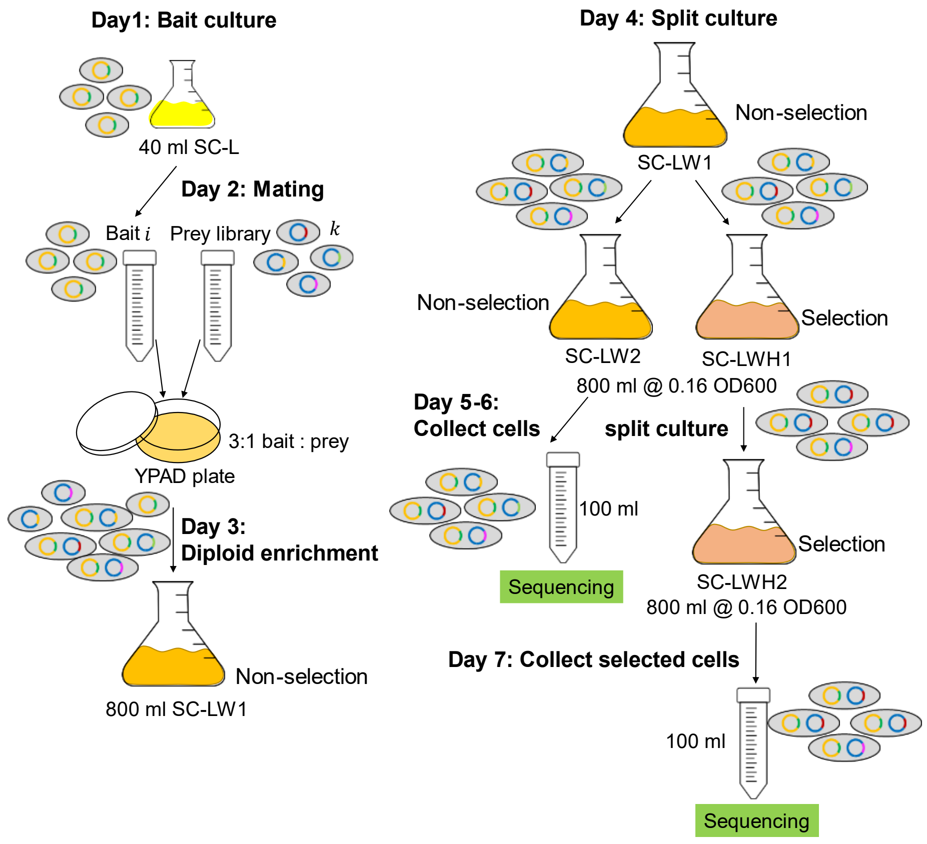

Supplies:
1. 50mL Falcon tubes
2. 12 x 250mL baffled flasks (autoclaved)
3. 25 x 2L baffled flasks
4. 2mL Eppendorf tubes
5. 1.5mL Eppendorf tubes
6. Cuvettes for Spec
7. 10uL pipette tips (prefer filter)
8. 200uL pipette tips (prefer filter)
9. 1mL pipette tips (prefer filter)
10. 5mL pipette tips
11. 10mL pipettes
12. 25mL pipettes
13. Cell scrapers (autoclaved)

Solutions:
1. 1L Sterile RO H_2O for washing
2. 2$^+$L Sterile 0.9% NaCl for washing
3. 10% bleach
4. 70% ethanol

Media:
1. 2$^+$L 40% Dextrose - DAYS1-6
2. 2$^+$L 10mM Adenine - DAYS1-6
3. 500mL SC-L liquid - DAY1
   a. 12 x 40mL bait cultures
4. 1L YPAD agar - DAY2
   a. 12 x 150mm YPAD agar plates
5. 800mL SC- agar - DAY3
   a. 36 x 100mm SC-LW agar plates
6. 12 x 800mL SC-LW^1 (2L glass baffled flasks) - DAY3
7. 1L SC-LWH liquid for washing - DAY4
8. 1 x 800mL SC-LW^2 (2L glass baffled flask) - DAY4
9. 12 x 800mL SC-LWH^1 (2L glass baffled flasks) - DAY4
10. 12 x 800mL SC-LWH^2 (2L glass baffled flasks) - DAY5-6

A note on media preparation - Make SC$^-$ in 4L or 4.8L batches:

4.8L SC$^-$ -> 6 x 800mL 2L flasks
4.8L SC$^-$ -> 6 x 800mL 2L flasks
} For DAY3 12 x 800mL SC-LW^1 flasks

4.8L SC$^-$ -> 6 x 800mL 2L flasks
4.8L SC$^-$ -> 6 x 800mL 2L flasks
} For DAY4 12 x 800mL SC-LWH^1 flasks

4.0L SC$^-$ ->
- 1 x 500mL SC$^-$ liquid in bottle (for DAY1 SC-L 40mL bait cultures)
- 1 x 800mL SC$^-$ agar in bottle (for DAY3 SC-LW 100mm plates)
- 1 x 800mL SC$^-$ liquid in 2L flask (for DAY4 SC-LW^2 flask)
- 1 x 1000mL SC$^-$ liquid in bottle (for DAY4 washing)
- 1 x 800mL SC$^-$ liquid in bottle for dilutions and OD monitoring

4.8L SC$^-$ -> 6 x 800mL 2L flasks
4.8L SC$^-$ -> 6 x 800mL 2L flasks
} For DAY6 12 x 800mL SC-LWH^2 flasks

Day -2: Start bait cultures on agar plates.

Date = ____________

1. Inoculate SC-L agar plate with Y8930 (p97-DB) bait strains from -80°C glycerol stock
   a. Streak for isolation of single colonies and incubate ~2-3 days at 30°C
2. Ensure all supplies are on hand and prepare all media and solutions except [12. 12 x 800mL SC-LWH[2] (2L glass baffled flasks) - DAY5-6] which will be prepared on DAY4 using SC-LWH[1] flasks

Day 1 (SAT): Start overnight bait strain liquid cultures. (start @ ~6:00pm)

Date = ____________

3. Pick 1-3 single colonies into 40mL of SC-L liquid media [final OD600 = ~**0.02** (~0.01 - .05 acceptable)]
   a. Grow overnight at 30°C 225 rpm (~14-18hrs to reach OD_{600} = ~3)

   Time = ___________

culture #	Colony Resuspend OD_{600} 10^{-1}	culture volume for 40mL @ 0.02 OD_{600}	SC-L volume for 40mL @ 0.02 OD_{600}	Over-night OD_{600} 10^{-1} Time: ______

Day 2 (SUN): Set up yeast mating. (start @ ~8-10:00am)

Date = ____________

4. Determine overnight culture titer using spectrophotometer.
   a. $OD_{600} = 10^{-1}$ (100uL cells in 900uL SC-L)
5. When bait cultures are ready (>2.5 OD_{600}), remove 12-13 aliquots of prey library from the -80°C and thaw on ice ~15mins.
6. Centrifuge 40mL bait cultures 1000g 5min in sterile 50mL tubes.

   Time = ___________
7. Discard supernatant and resuspend pellets in 5mL sterile H_2O.
8. Determine titer using spectrophotometer and 1:100 dilution.
   a. $OD_{600} = 10^{-2}$ (10uL cells in 990uL H_2O)
   b. Expected range: OD_{600} 10^{-2} = ~0.15-0.2
9. Combine prey aliquots in a 50mL tube, gently mix, and re-aliquot 1 mL to 2mL tubes.
10. Add appropriate bait volume (step #8) to each labeled prey tube.
11. Mix gently and centrifuge 1000g 5min. Remove supernatant.
12. Resuspend pellet in 1mL sterile H_2O
13. Incubate mating tubes on sides for ~3hr @ ~100-115rpm @ 30°C.

    Time = ___________
14. Pour twelve 150mm YPAD agar plates and leave covered in hood to solidify until ready to use (~3hrs).
15. After 3hr incubation, centrifuge 1000g 5min. Remove supernatant.

    Time = ___________
16. Resuspend pellet in 500uL sterile H_2O.
17. Plate cells on 150mm YPAD agar plates with 6-8 4mm glass beads to spread.
18. Wash tubes with 250uL sterile H2O and add to plates. Shake beads to spread.

Subculture culture #	OD_{600} 10^{-2}	volume for 1.8×10^8 cells

19. Let plates air dry in hood ~10-15mins until surface is dry. Incubate inverted 16-18hrs @ 30°C.
    Time = ____________

Day 3 (MON): Collect cells and start liquid cultures for diploid enrichment. (start @ ~10:00am-12:00pm)

Date = _______________

20. Prepare diploid enrichment flasks and dilution plates/tubes for post-mating titering:
    a. 12 x SC-LW 800mL (final): Add 45mL 40% Dextrose, 45mL 20mM Adenine, and 6.8mL 100mM Histidine
        i. Dextrose, Adenine and Histidine can be added to flasks during wash steps #22-25
    b. SC-LW agar plates - 3 plates (10^{-3}, 10^{-4} and 10^{-5}) per mating - label with bait # and dilution on frosted part
        i. Add 6-8 3mm glass beads to each plate
    c. Dilution tubes (1.5mL) with sterile 0.9% NaCl:
        i. 10^{-2} (990uL), 10^{-3} (900uL), 10^{-4} (900uL), 10^{-5} (900uL)
21. Collect cells from YPAD mating plates **Time = ____________**
    a. Pipet 5mL 0.9% NaCl onto plate and use cell scraper to gently dislodge cells.
    b. Tilt plates to collect cell solution and add to 50mL labeled tube
    c. Repeat to wash plate 4 times with 5mL 0.9% NaCl (total volume = 25mL)

<u>NOTE</u>: These cells are actively respiring and CO_2 can build up within the tube. Crack the lid of each tube while processing the other samples. Be sure to swirl a little bit and then tighten the lid before centrifuge steps. Leave the tubes slightly cracked and gently swirl before capping tightly and gently vortexing to resuspend the pellet.

22. Vortex low and centrifuge 50mL tubes 2000g 20min. Remove supernatant.
23. Wash 1: Resuspend pellet with 25mL 0.9% NaCl. Vortex low and centrifuge 50mL tubes 2000g 15min. Remove supernatant.
24. Wash 2: Resuspend pellet with 25mL 0.9% NaCl. Vortex low and centrifuge 50mL tubes 2000g 15min. Remove supernatant.
25. Wash 3: Resuspend pellet with 25mL 0.9% NaCl. Vortex low and centrifuge 50mL tubes 2000g 10min. Remove supernatant.
26. Resuspend pellet with 23.5mL 0.9% NaCl (~25mL final).
27. Remove 10uL sample and resuspend in 10^{-2} dilution tube.
28. Estimate cell titer using spectrophotometer.
    a. $OD_{600} = 10^{-2}$
    b. Use 700uL for OD measurements to leave enough for further dilutions.
    c. Expect range: OD_{600} 10^{-2} = ~0.10-0.12
29. Add cells to 800mL SC-LW media in glass baffled flasks.
30. Wash tubes with 25mL SC-LWH and add to flasks.
31. Start culture incubations (225rpm @ 30°C for ~18hrs).
    Time = ____________
32. Perform dilution plating:
    a. Vortex tubes prior to dilutions and plating.

culture #	$OD_{600} = 10^{-2}$	est. cell OD_{600}	estimated cell titer (25mL total volume)

b. 10^0 (step #27) —10uL→ 10^{-2} —100uL→ 10^{-3} —100uL→ 10^{-4} —100uL→ 10^{-5}

c. Plate 100ul of the appropriate dilution on 10^{-3}, 10^{-4} and 10^{-5} plates and spread with glass beads

Day 4 (TUES): Check OD_{600} (start @ ~8:00-10:00am) and subculture into SC-LW and SC-LWH flasks.

Date = ____________

33. Prepare 10^{-1} (900uL SC-LWH) and 10^{-2} (990uL SC-LWH) dilution tubes for each bait and label cuvettes.
34. During centrifuge steps #38-42, prepare media flasks:
    a. 1 x SC-LW[2] 800mL (Non-selected condition)
        i. Add 40mL 40% Dextrose, 40mL 20mM Adenine, and 6.5mL 100mM Histidine
    b. 12 x SC-LWH[1] 800mL (Selected condition, 1st round)
        i. Add 40mL 40% Dextrose and 40mL 20mM Adenine
35. Determine overnight SC-LW culture titer using spectrophotometer.
    a. $OD_{600} = 10^{-1}$ (100uL cells in 900uL SC-LWH)
    b. Expect OD_{600} 10^{-1} = ~0.25-0.35 after 16-18 hours

Check #1: Time = __________

culture #	OD_{600} 10^{-1}

Check #2: Time = __________

culture #	OD_{600} 10^{-1}

Check #3: Time = __________

culture #	OD_{600} 10^{-1}

36. Once cultures reach OD_{600} 10^{-1} > 0.25, harvest cells.
37. Mix culture well and pour/pipette 50mL into a labeled Falcon tube.
38. Centrifuge cells 2000g 10min. Remove supernatant.
39. Mix culture well and pour/pipette 50mL into the same tube for a total of 100mL cell culture harvested.
40. Centrifuge cells 2000g 10min. Remove supernatant.
41. Wash 1: Resuspend pellet with 25mL SC-LWH. Vortex low and centrifuge 50mL tubes 2000g 5min. Remove supernatant.
42. Wash 2: Resuspend pellet with 25mL SC-LWH. Vortex low and centrifuge 50mL tubes 2000g 5min. Remove supernatant.
43. Resuspend pellet in 25mL SC-LWH.
44. Check $OD_{600} = 10^{-2}$ (10uL cells in 990uL SC-LWH)

Subculture Cells: Time = _______

Step #44 Washed and resuspended culture #	OD_{600} 10^{-2}	culture volume for ~800mL @ 0.16 OD_{600}	Select random bait for Additional SC-LW[2] culture

45. Subculture enough cells to start new 800ml SC-LW and SC-LWH cultures @ 0.16 OD_{600}.
    a. Volume to add = (800*0.16)/(OD_{600} step #48)/2
46. Select one bait at random for the non-selected SC-LW culture.
47. Start culture incubations **Time = ___________**
    a. Incubate SC-LW flask (225rpm @ 30°C) for ~16-18 hours
    b. Incubate SC-LWH flasks (225rpm @ 30°C) for ~60-74 hours
48. Autoclave SC-LWH[1] flasks, wash, and prepare 12 x SC- 800mL for DAY5-6.

Day 5 (WED): Check OD_{600} (start @ ~7:00-8:00am) and harvest SC-LW[2].

Date = ______________

49. Determine SC-LW[2] culture titer using spectrophotometer.
    a. OD_{600} = 10^{-1} (100uL cells in 900ul SC-LWH)
    b. Expect OD_{600} 10^{-1} = ~0.25-0.35 after 16-18 hours

Date = __________

Check #1: Time = __________

SC-LW[2] culture #	OD_{600} 10^{-1}	Harvest?

Date = __________

Check #2: Time = __________

SC-LW[2] culture #	OD_{600} 10^{-1}	Harvest?

Date = _________

Check #3: Time = __________

SC-LW[2] culture #	OD_{600} 10^{-1}	Harvest?

50. Once SC-LW[2] cultures reach OD_{600} 10^{-1} > 0.25, harvest 2 x 100mL samples. **Time = ___________**
    a. Label two 50mL Falcon tubes per sample with experiment #, “SC=LW”, bait#, “A” or “B”, and date
    b. Mix culture well and pour 50mL into a labeled Falcon tube.
    c. Centrifuge cells 3000g 5min. Remove supernatant.
    d. Mix culture well and pour additional 50mL into Falcon tube.
    e. Centrifuge cells 3000g 5min. Remove supernatant.
    f. Invert tubes on a paper towel to remove residual media and then freeze tubes at -20°C.

Days 6-7 (THUR/FRI): Check OD_{600} of SC-LWH^1 culture periodically and subculture once OD_{600} $10^{-1} > 0.25$.

51. Determine SC-LWH^1 culture titer using spectrophotometer.
    a. $OD_{600} = 10^{-1}$(100uL cells in 900ul SC-LWH)
    b. Expect OD_{600} 10^{-1} = ~0.25-0.30 after ~60-72 hours

Date = __________
Check #1: Time = __________

SC-LWH^1 culture #	OD_{600} 10^{-1}	culture volume for ~800mL @ 0.16 OD_{600}

Date = __________
Check #2: Time = __________

SC-LWH^1 culture #	OD_{600} 10^{-1}	culture volume for ~800mL @ 0.16 OD_{600}

Date = _________
Check #3: Time = _

SC-LWH^1 culture #	OD_{600} 10^{-1}	culture volume for ~800mL @ 0.16 OD_{600}

Date = __________
Check #4: Time = __________

SC-LWH^1 culture #	OD_{600} 10^{-1}	culture volume for ~800mL @ 0.16 OD_{600}

Date = __________
Check #5: Time = __________

SC-LWH^1 culture #	OD_{600} 10^{-1}	culture volume for ~800mL @ 0.16 OD_{600}

Date = __________
Check #6: Time = __________

SC-LWH^1 culture #	OD_{600} 10^{-1}	culture volume for ~800mL @ 0.16 OD_{600}

52. Once cultures reach OD_{600} $10^{-1} > 0.25$, subculture cells into fresh 800mL SC-LWH^2 culture.
    a. Prepare SC-LWH^2 culture flasks: Add 40mL 40% Dextrose and 40mL 20mM Adenine
    b. Mix culture well and pipet enough cells into SC-LWH^2 flasks to reach OD_{600} = ~0.16
    c. Incubate SC-LWH^2 flasks in Wise lab shaker (225rpm @ 30°C) for ~18-24 hours.
    Time = ___________

Days 7-8 (FRI/SAT): Check OD_{600} of SC-LWH^2 culture periodically and harvest cells once OD_{600} $10^{-1} > 0.25$.

Date = ___________

53. Determine SC-LWH^2 culture titer using spectrophotometer.
    a. $OD_{600} = 10^{-1}$ (100uL cells in 900ul SC-LWH)
    b. Expect OD_{600} 10^{-1} = ~0.25-0.35 after ~18-24 hours

Date = __________

Check #1: Time = __________

SC-LWH^2 culture #	OD_{600} 10^{-1}	Harvest?

Date = __________

Check #2: Time = __________

SC-LWH^2 culture #	OD_{600} 10^{-1}	Harvest?

Date = _________

Check #3: Time = __________

SC-LWH^2 culture #	OD_{600} 10^{-1}	Harvest?

54. Once SC-LWH^2 cultures reach OD_{600} $10^{-1} > 0.25$, harvest 2 x 100mL samples.
    a. Label two 50mL Falcon tubes per sample with experiment #, “SC=LWH”, bait#, “A” or “B”, and date
    b. Mix culture well and pour 50mL into a labeled Falcon tube.
    c. Centrifuge cells 3000g 5min. Remove supernatant.
    d. Mix culture well and pour additional 50mL into Falcon tube.
    e. Centrifuge cells 3000g 5min. Remove supernatant.
    f. Invert tubes on a paper towel to remove residual media and then freeze tubes at -20°C.
55. DONE!
