# Supplementary material for: Next-generation yeast-two-hybrid analysis with Y2H-SCORES identifies novel interactors of the MLA immune receptor
Source: PLoS Comput Biol. 2021 Apr 2;17(4):e1008890. doi: 10.1371/journal.pcbi.1008890 (PMC8046355; doi:10.1371/journal.pcbi.1008890)
Supplement: Attachment — Submitted filename: Velasquez Response to reviewers.pdf [file pcbi.1008890.s020.pdf]

OVERVIEW – Velasquez-Zapata et al.

We have received and assessed the comments and corrections on our manuscript "Y2H-SCORES: A statistical framework to infer protein-protein interactions from next-generation yeast-two-hybrid sequence data".

All comments were very constructive and helped us to produce a fully improved version. Importantly, we now show the broad applicability of our method to diverse experimental designs and Y2H systems. Furthermore, we demonstrate our scoring system performs as well as or better than previously published approaches. In addition, we now include a complete dataset that we used to identify interactions between the plant disease resistance protein MLA6 and novel proteins involved in plant immune response, which should be of great interest to the plant-microbe interactions community. Taken together, we are confident that the revised manuscript satisfies reviewers' comments and will impact researchers probing protein-protein interactions at small and large scales, researchers involved in reconstructing protein interactome networks, and researchers interested in plant NLR biology.

Briefly, we have benchmarked our method using five diverse Y2H-NGIS studies which had count data available. We compared the performance of each of the three scores proposed in our manuscript and a Borda ensemble with each reference method. We also have included a validation dataset consisting of 3 bait screenings for 3 fragments of the barley immune receptor, MLA6. This validation dataset contains 17 confirmed positive interactions and 38 negative tests across a range of confidence scores. The results from this dataset allows users to set a cut-off for the parameters of Y2H-SCORES that provides high confidence of a positive interaction. Furthermore, we have considerably rewritten the manuscript, particularly key paragraphs in the Introduction, Results and Discussion sections, in which we have clarified some of our approaches, interpretations and conclusions. We have made available all the data and code necessary to reproduce our analysis which can be found at https://github.com/Wiselab2/Y2H-SCORES/tree/master/Publication, and the Y2H-SCORES software is now available at https://github.com/Wiselab2/Y2H-SCORES/tree/master/Software

We are confident that this thoroughly revised and further focused manuscript is more rigorous and suitable given the scope of the research topic of the journal. Finally, we want to extend our thanks to the reviewers for taking the time and effort to provide such insightful comments, and the opportunity to review and resubmit our work.

RESPONSES TO REVIEWERS

Reviewer #1: Here in this paper, authors proposed the novel method to analyze yeast two-hybrid data based on next generation sequencing of cDNA library-constructed prey pool. However, considering the recent (last five years) progress of NGS-based Y2H with fused ORFs/barcodes and constructed individually cloned library of ORFs, such as SynAg (Younger et al., 2017), BFG-Y2H (Yachie et al., 2016), PPiSeq (Schlecht et al., 2017), BI-Tag (Hastie and Pruitt, 2007), iCLIP (Nirantar and Ghadessy, 2011), CrY2H-seq (Trigg et al., 2017), Rec-YnH (Yang et al., 2018), and RLL-Y2H (Yang et al., 2018), I think this topic is a little bit outdated. It will be great if this method is shown to be applicable to these novel methods.

Authors response: Thank you so much for pointing to these references. Some were already cited by us, others were of a great help to benchmark our method. We could use the count data reported by BFG-Y2H (Yachie et al., 2016), PPiSeq (Schlecht et al., 2017), Rec-YnH (Yang et al., 2018), DEEPN (Pashkova et al., 2016) and the raw data supplied by Erffelinck et al., (2018) to compute Y2H-SCORES and compare its performance to the reference methods. The results indicate that Y2H-SCORES performs as well as or superior to the scoring implemented in these studies, demonstrating that Y2H-SCORES can be broadly implemented under a range of experimental systems and should be useful for any researcher

performing Y2H-based interactome mapping. These results can be found in the manuscript section "Y2H-SCORES can be implemented with different yeast-based interaction screenings"

Reviewer #2: The authors described Y2H-SCORES, a statistical framework to infer protein-protein interactions from next-generation yeast-two-hybrid sequence data also referred as Y2H-NGIS.

Y2H-NGIS appeared to be a powerful approach to dissect protein-protein interactions and will undoubtedly become a standard in the fields of interactomics along with other methods such as AP-MS experiments. The authors benchmark the Y2H-SCORES using experimental and simulated datasets and validate experimentally their scoring system on one Y2H-NGIS screen. Y2H-SCORES is used in combination with NGPINT which process Y2H-NGIS raw data sequences. Y2H-SCORES and NGPINT are in some extent dependent and are developed by the same authors. The two algorithms have been submitted at the same time and separately on bioarxiv.

Major revisions:

1) The authors need to cite published tools that are available to analyze Y2H-NGIS. They have to compare Y2H-SCORES to the state-of-the-art algorithms (DEEPN, etc.);

Authors response: This is a common concern for all the reviewers. Acquisition of raw data from other Y2H-NGIS studies was a challenging task – during our study, we had contacted several investigators multiple times with no response. However, after reviewing more references (including those cited by reviewer #1, we found that we could use several count datasets reported in supplemental materials to benchmark our method. Among them we analyzed BFG-Y2H (Yachie et al., 2016), PPiSeq (Schlecht et al., 2017), Rec-YnH (Yang et al., 2018), DEEPN (Pashkova et al., 2016) and Erffelinck et al., (2018). These results can be found in the manuscript section "Y2H-SCORES can be implemented with different yeast-based interaction screenings"

2) The authors validate their algorithm on simulated data. As the simulator is in some extent an original development, it might be crucial to make the source code available for reproducible research;

Authors response: Thank you for your comment on this. Yes, the simulator is a new development because there is no available simulators for these types of data. We updated the GitHub page for the publication and added the code for the simulator at https://github.com/Wiselab2/Y2H-SCORES/tree/master/Publication/Y2H-NGIS_simulator.

3) The authors state that "it is nearly impossible to compare Y2H-NGIS studies". As some Y2H-NGIS might be available in open access archives, the authors should demonstrate that their algorithm might be able to reproduce results already published (true positive and false positive) and to make these other studies comparable.

Authors response: We initially made this claim because each method has its own experimental settings and performance values should only be analyzed in the context of each method. Please see our replies to Reviewer #1 and Reviewer #2.1. We now demonstrate that Y2H-SCORES has a similar or superior performance than the cited methods, despite the major differences among them. Results in the manuscript section "Y2H-SCORES can be implemented with different yeast-based interaction screenings" show how Y2H-SCORES adapts to different Y2H-NGIS datasets. Using simulation and our experimental dataset with MLA6 baits we demonstrated that the performance of Y2H-SCORES can be improved by the experimental setup and control of different variables, thus researchers that are new to this field have a set of guidelines or best practices that can be implemented for successful Y2H-NGIS.

4) The authors did not discuss the false negative rate in Y2H-NGIS. To complete their work, the authors should use Y2H-SCORES to evaluate experimentally the sensitivity of the experiments by using independent Y2H-NGIS batches. In addition, the authors need to explain if the method is able to provide enough sensitivity to detect all the partners of hub proteins, those protein having potentially more than 100 known partners depending of their biological context.

Authors response: Thank you for highlighting this important aspect which was not discussed in the manuscript. We have now included discussion of false negative rates in Y2H which may influence this molecular technique to identify an interaction (see lines 410-417, 630-631 in the manuscript). For this purpose, we used the dataset from Pashkova et al., 2016, which contains ubiquitin and Rab5 as baits and a large validation set reported in BioGRID, giving us a better idea of the number of false negatives. These proteins, considered as hubs, were analyzed by Y2H-SCORES, and the favorable performance of the method and this reference demonstrates that Y2H-NGIS is useful for identifying interactors from hub proteins, as long as they are identified by Y2H. We also now include a complete dataset of validated interactions with the NLR MLA6, and demonstrate that our scoring system can confidently predict true interactors from Y2H-NGIS data. We hope the reviewer can understand how difficult it would be to obtain a true measurement of the false negative rate within the data, which would require tens of thousands of one-to-one binary tests, not to mention the associated cloning of all the genes for testing.

5) The authors use different baits and Y2H-NGIS to benchmark the normalization steps and the scoring functions of the Y2H-SCORES. To make their study reproducible, it is compulsory that the raw data (.fastq) will be made available in SRA or ENA archive;

Authors response: We have made available the raw reads in GSE164814 (MLA61-161), GSE164815 (MLA61-225), GSE164816 (MLA6550-956), GSE164761 (luciferase - construct #1) and GSE164762 (luciferase - construct #2). The total count dataset for the baits that were used to assess normalization is located in S1 Table; fusion counts can be found in S10 Table. These tables were used to run Y2H-SCORES and experimentally validate its performance. Results can be found in the manuscript sections "The effect of normalization on Y2H-NGIS data" (lines 159-241) and "Experimental validation of Y2H-SCORES identifies novel interactors of the MLA6 barley powdery mildew resistance protein" (lines 440-495).

6) The results of the Y2H-SCORES use in the case study are not available. The authors have to provide raw result tables as well a selection of bona fide candidates in Supplementary files;

Authors response: Please see our response to the previous comment. In addition, the full Y2H-SCORES table for the MLA6 baits is now available in S8 Table. We also report a total of 17 positive and 38 negative interactions in this dataset. This table contains each of the three scores and the Borda ensemble. The column "interaction" shows the experimental validation, with the bona fide interactions assigned with a value of 1. Raw total and fusion counts are reported in S1 and S10 tables.

Minor revisions:

1) The authors state "the massive and diverse datasets resulting from this technology". I am not convinced that the number of publications using this technology is large enough to be considered as "massive". The authors should review how many Y2H-NGIS datasets are available and add references if possible.

Authors response: We agree with the reviewer in that the number of references in this topic is not massive yet. We were referring more to the amount on information that can be found in one screen, since it is high throughput. We changed the term massive from the summary and added references as possible to highlight the potential of Y2H-NGIS. Thank you for including more Y2H-NGIS references in a previous comment. We did indeed review some of those and now include some that were overlooked in our first version. We also discuss how these datasets differ from one another in sections/lines 378-436.

2) The authors show that 5 replicates provide better result in term of specificity. It is important to explain why the authors decided to launch their case study with only three replicates.

Authors response: The number of replicates in the experiment was chosen based on time and financial constraints. Simulation showed no difference in ROC AUC and showed little difference in the PR AUC values between 5 and 3 replicates. The greatest difference in PR AUC was found for the enrichment score going from 0.62 to 0.65, which makes 3 also a valid option to optimize screening resources.

Reviewer #3: This study proposes a statistical approach for ranking protein-protein interactions (PPIs) by analyzing yeast two-hybrid integrated with next generation sequencing (Y2H-NGIS) data. The proposed approach relies on existing work that has mapped and quantified raw reads from Y2H-NGIS data and thus provided both total and fusion prey counts under selective and non-selective conditions. Then, the authors' approach ranks candidate prey-bait interactions based on these count data. Inference of correct PPI network data is an important problem, as such data remains incomplete for most species, including yeast (the simplest eucaryotic model organism) and human (the organisms most relevant for studying and understanding disease).

The writing of the paper needs improvement in terms of both content clarity and grammar.

Regarding content clarity, here are just some examples of phrases/sentences are unclear (they key issue is that the paper isn't adjusted to each of biologists, statisticians, and computational scientists who study biological networks, but all of whom are relevant audience for this paper):
- I don't understand this sentence: "[framework] for analyzing high-throughput Y2H screens that considers key aspects of experimental design, normalization, and controls."

Authors response: Thank you for your comments. We are aware that this topic is in an intersection of different scientific fields which can make it difficult to understand. We have rewritten several sections in the paper to make them more understandable to a broad audience. We reorganized the figures to introduce the topic better, showing the experimental workflow of the technique first, as is shown in Fig 1. We also added general definitions of Y2H in lines 99-105.

We changed the sentence "[framework] for analyzing high-throughput Y2H screens that considers key aspects of experimental design, normalization, and controls." by “Here, we present a computational framework, designated Y2H-SCORES, for analyzing high-throughput Y2H screens. Y2H-SCORES considers key aspects of NGIS experimental design and important characteristics of the resulting data that distinguish it from RNA-seq expression datasets.” See lines 56-59.

In the introduction (lines 145-148) we also added more descriptions of what Y2H-SCORES does: “Here, we propose statistical methods to analyze Y2H-NGIS count data and rank the resulting prey candidates into a high-confidence list of interactors. Given prey total and fusion count tables for a bait screen, we calculate a set of three scores to rank candidate bait-interactors, designated Y2H-SCORES ”.

- The description of the three scores (including why there are three scores and why these particular three scores) on lines 48-51 as well as throughout the paper is unclear. These scores are mentioned in abstract, intro, results, discussion, methods, i.e., at least 4 times, but each time, almost the exact same text is stated, which is very brief and for me non-informative. Please clearly explain each score and motivate the three scores.

Authors response: We used consistent terminology to explain the scores to make it easier for the reader to understand. We explained the scores further and referenced one publication that had discussed the concepts of enrichment and specificity in Y2H (Suter et.al, 2015). The three scores are related with biological properties of true interactors in Y2H which we explain in more detail at lines 246-252. The novelty of our method, among other aspects of the paper, was to develop a quantitative measure of those properties. To further clarify, our approach, we created a figure (panel A of Fig 3) which illustrates the basis of the three scores. We hope this provides more clarification about how the scores work.

- "Simulation of Y2H-NGIS identified"?

Authors response: reworded to "Simulation of Y2H-NGIS data enabled us to identify"

- Why Introduction opens up with and motivates the study mostly by discussing host-pathogen interactions when interactomes are critical for numerous applications of network biology?

Authors response: We introduced this topic focusing of host-pathogen interactions because that was the context of the experimental dataset that we used to validate the method. This comment helped us to reorganize the text so the Introduction is more general and the Discussion concludes with results obtained with the MLA6 bait, a barley NLR resistance protein involved in plant immune responses.

- "the result of Y2H screens"?

Authors response: we rephrased to "candidate interactors obtained from the Y2H screens"

- Why studying total reads vs. prey-fusion reads is an issue.

Authors response: Thank you for this comment. This is a very important distinction in the manuscript, which we should make as clear as possible. We added more details on the benefits of using both types of reads to score interactions, especially from datasets that use cDNA prey libraries in contrast to ORF libraries (lines 119-126).

From the new introduction: "Currently available pipelines map and quantify total reads while ignoring prey-fusion reads (17,18), or map fusion reads without quantifying their biological significance to identify interactions (19). Fusion reads contain both Y2H plasmid and prey cDNA sequence, and contain details on the translational fusion of the prey sequence with the TF activation domain in the hybrid protein. This information is useful to verify if the cDNA fragment is in frame with the TF activation domain, and thus, the native peptide sequence is expressed in yeast. ORF libraries do not have this issue because they are assumed to be all in frame with the prey plasmid; however, ORF prey libraries are only available for few model organisms (5)."

- On lines 104-124, provide appropriate references under each of points 1-4, so that it's clear, for each reference (i.e., each existing approach for the same or similar purpose as the proposed approach), which of the drawbacks 1-4 it has. That is, are there any existing approaches that do not have all 4 drawbacks?

Also, what are all relevant existing approaches? These need to be discussed. In one place, references 10-15 are cited, but in another place, 11-14, 16, and 18 are cited. So are all of 10-18 relevant existing methods?

Authors response: We have included more references to the list to make it more complete. As we now explain in the text starting at line 113, there is no published approach that does not have all issues we cite. Most of the references are studies that apply Y2H-NGIS to answer a biological question, they don't offer an extensive discussion of the data analysis nor offer software. Therefore, they are relevant since they use Y2H-NGIS, but their ranking methods cannot be widely used to analyze Y2H-NGIS data. Only three references offer software (Pashkova et.al., 2016; Yang et at., 2018 and Trigg et al., 2017), but each of them has disadvantages, which are explained in the introduction on lines 119-120: "Currently available pipelines map and quantify total reads while ignoring prey-fusion reads, or map fusion reads without quantifying their biological significance to identify interactions". One novel aspect of our analytical method is to present a flexible software that account and compensate for the identified issues.

- The statement "Hence, there is a need for robust and consistent statistical models that make use of all the available information in Y2H-NGIS data." should be moved way up in the text, as the motivation for the discussion that is currently prior to this sentence.

Authors response: Thanks for this comment. The reviewer is right, and we are now including this clear motivation for our study in the introduction on lines 113-117. The phrase was moved to the beginning of the paragraph, so it is obvious to readers.

- "Principal component analysis (PCA) of the log-transformed Y2H-NGIS raw read counts identified selection as the major source of variability": Why is PCA done? What question is being asked? This sentence seems misplaced, its purpose is unclear. Even its meaning is unclear, e.g., what THE log-transformed read counts, from which data type and data source? What does "identified selection" mean? What kind of variability?

Authors response: We appreciate this comment because we realized that we could more clearly articulate the motivation behind the analysis we performed in this section. We added a description at the beginning of the section on normalization explaining the type of data and the motivation of the analysis (lines 175-183): We tested different normalization methods for their ability to reduce the inter-replicate variability of Y2H-NGIS counts obtained from NGPINT, while maintaining other sources of biological variation, including the selection condition and bait identity. Principal component analysis (PCA) of the Y2H-NGIS raw read counts (S1 Table) was applied to evaluate changes in the variability of raw and normalized counts.

- "interactors should grow exponentially"?
- "non-interactors should not reproduce under selection"?
- "non-interactors should not reproduce under selection. Our goal is to identify prey whose relative abundance in the selected samples increases over the relative abundance in the non-selected samples"?
- And so on... I ask that the authors carefully think how to describe their study to someone interested in analyzing and even predicting PPI networks but who might not be an immediate expert in the specific task of scoring PPIs from Y2H data.

Authors response: These sentences are associated with understanding how Y2H identifies protein-protein interactions. We now include a more thorough explanation in the introduction as to how Y2H

works and why only true interactors should grow under selection (lines 99-105). We also added a description of the experimental setting at the beginning of the results section (lines 159-168) followed by Fig 1 with a graphical summary of the process, We expect this provides more information about the basic idea behind Y2H-NGIS and why only bait interactors should reproduce under selection.

Regarding grammar:
- There is often mix of singular and plural (e.g., on line 41, models is plural but the organism scale is singular, and there is only one interactome/model per organism, no?; on line 83, investigations ... has benefited; on line 93, it says THE screen -- which THE screen? -- while on line 96 it says screens; prey in line 168 -- I believe this should be preys; and so on).

Authors response: We thank the reviewer for pointing this out, we have rephrased these sentences to fix inconsistencies.

- There is some text redundancy (e.g., on lines 41 and 42, models -- i.e., interactomes -- and interacting pairs of proteins mean the same thing).

Authors response: Thank you, we addressed this comment by removing "interacting pairs of proteins" from the text.

- There is inconsistent use of terminology (e.g., the interactome vs. gene/protein networks vs. signaling networks vs. protein interactome networks vs. protein-protein interaction networks vs. Protein-Protein Interaction networks vs. PPI networks ...).

Authors response: Thank you for pointing this out - we have now unified the terminology and refer to interactome networks as the collection of protein-protein interactions (PPI).

- line 85: "this knowledge": which knowledge?

Authors response: As part of our revisions, this phrase was removed from the paper.

Possibly because of writing issues, I am unclear what the problem statement of this paper is: given what as input, what is the output of the proposed method, and what is the goal (e.g., to optimize what objective function)? This needs to be clearly and precisely stated. Statements such as "interacting partners obtained from Y2H-NGIS, including mapping reads to the reference prey genome, reconstruction of prey fragments, and distinguishing fusions with the prey activation domain (20). Here, we propose statistical methods to rank the resulting preys, distinguish false vs. true interactors, providing to the user a high-confidence list of candidates." are unclear, i.e., they are not specific enough. I'm looking for something like: Given what data as input (represented as a matrix of dimensions a x b, where a is the number of genes/proteins and b is , ...), the goal is to score each pair of genes/proteins such that ..., and to do similar input-output discussion for each step of the methodology and evaluation. Current descriptions are vague as well as very terminology-specific, i.e., they are not detailed enough as well as not adjusted for scientists who might not be experts in Y2H data but who are experts in general biological network inference and analysis.

Authors response: Thanks for your comment. We highlighted the purposes of Y2H-SCORES and the difference with NGPINT in lines 145-148, 165-168 and 243-246. Summarizing, NGPINT processes Y2H-NGIS raw reads giving total and fusion counts as outputs; Y2H-SCORES takes counts from NGPINT or other mapping tools and computes 3 scores to rank the candidate interactors. We described the

purpose of each score in lines 243-252 and how they correlate with three biological properties (see response on the purpose of the scores).

We changed as "interacting partners obtained from Y2H-NGIS, including mapping reads to the reference prey genome, reconstruction of prey fragments, and distinguishing fusions with the prey activation domain (20). Here, we propose statistical methods to rank the resulting preys, distinguish false vs. true interactors, providing to the user a high-confidence list of candidates." to: "Here, we propose statistical methods to analyze Y2H-NGIS count data and rank the resulting prey candidates into a high-confidence list of interactors. Given prey total and fusion count tables for a bait screen, we calculate a set of three scores to rank candidate bait-interactors, designated Y2H-SCORES."

In addition, in the notation of the methods (lines 733-838 and S2 Text, S5 Fig), we explain what the data is and what each subindex represents. We also included S1 and S10 tables with the raw datasets, and the total scores in S5 and S9 Tables so the reader can see what those indexes in the math models represent.

I am unclear what the novelty of the proposed work is given the text on lines 126-145 as well as that the proposed approach relies on an existing study "that has [already] mapped and quantified raw reads from Y2H-NGIS data and thus provided both total and fusion prey counts under selective and non-selective conditions". Specifically, it needs to be very clear how this paper is different than all existing methods for inference of PPIs from Y2H data, and especially compared to references 11 and 20.

Authors response: We hope that with the new explanations starting at line 145, that the differences between Y2H-SCORES and NGPINT are clear (see previous response). NGPINT processes raw data and offers prey total and fusion counts as output. Y2H-SCORES processes those count datasets and then generates 3 scores to rank the candidate interactions. In the current revision Y2H-SCORES has been extended to work with count datasets from different studies, as demonstrated in the benchmarking section (line 376).

We also compared Y2H-SCORES to DEEPN, which also screened cDNA libraries to discover interactions. Despite the fact that DEEPN identifies fusion reads, they don't use them as evidence to rank interactors nor offer the fusion counts as outputs. We take the fusion prey counts generated by NGPINT and built a quantitative measure for the enrichment of *in-frame* preys under selection. Despite DEEPN offering a software (last updated 3 years ago) for analysis, in our hands it is non-functional, and despite our best efforts, we could not execute it to analyze our Y2H-NGIS dataset.

My possibly biggest comment is related to evaluation in this paper.
- Real gold standard PPI data should be used to benchmark the performance of the proposed approach rather than just simulated data, and the real gold standard data should be independent (e.g., resulting from different experiment types) than the data used by the authors to predict PPIs.

Authors response: This was a common concern of the reviewers and presented an opportunity to look further for datasets to benchmark Y2H-SCORES. After reviewing the references and considering that for running Y2H-SCORES we only need count datasets and not raw data, we used the count data reported by BFG-Y2H (Yachie et al., 2016), PPiSeq (Schlecht et al., 2017), Rec-YnH (Yang et al., 2018), DEEPN (Pashkova et al., 2016) and the raw data supplied by Erffelinck et al., (2018) to compute Y2H-SCORES and compare its performance to the reference methods. As all these datasets contained baits with previously reported gold-standard interactions in BioGRID, we used this database in conjunction with experimental validation from each reference to calculate the performance.

There were other datasets that we requested over the years to test our software but they were not shared by the authors. Because many datasets associated with manuscripts that used similar

approaches did not report publicly available accession numbers, we wrote to four separate groups (Zong et al., 2020; Kessens et al., 2018; Pashkova et al., 2016; Erffelinck et al., 2018). This was to show proof of concept for the NGPINT front-end software. Only the Goossens group https://journals.plos.org/plosone/article?id=10.1371/journal.pone.0201270 responded and provided data which was used to test the NGPINT pipeline (now published in Briefings in Bioinformatics https://doi.org/10.1093/bib/bbaa351). We took the outputs from NGPINT with this dataset to run Y2H-SCORES. The results can be found in the benchmarking section (line 376).

We also performed our own original Y2H-NGIS experiment (deposited at NCBI-GEO GSE164814 (MLA61-161), GSE164815 (MLA61-225), GSE164816 (MLA6550-956).

- Regarding simulated data, I'm not entirely clear what true vs. false interactions are (these are briefly defined but to me these definitions are unclear -- another writing issue). Based on the current description, it seems that the authors are defining true vs. false interactions from the same data that they use to predict/rank/score their PPIs. If this is the case, there is a circular argument.

Authors response: The concept of true interactors was used before us by Yachie et al 2016. We added this reference to the text when we more clearly define these terms (lines 253-257). This concept refers to a prey that interacts specifically with a bait (i.e., it is not auto-active).

We defined the simulation in terms of true and false interactors as this is a key concept in the dynamics of the yeast growth during Y2H. We expect that true interactors grow faster than false interactors during selection since they activate the reporter gene (and our empirical results showed this as well). We represented them in the simulation model by sampling the fitness parameter from the top percentiles of the distribution. True and false interactors represent the ground truth in the validation set used to calculate the performance of the method during the simulation. S2 text has all the simulation details and the differences in true and false interactors in this model.

- Comparison to existing recent and state-of-the-art approaches for the same or similar purpose must be done, in terms of both accuracy and running time.

Authors response: Thank you, please see our responses to reviewers #1 and #2.1. We could use the count data reported by BFG-Y2H (Yachie et al., 2016), PPiSeq (Schlecht et al., 2017), Rec-YnH (Yang et al., 2018), DEEPN (Pashkova et al., 2016) and the raw data supplied by Erffelinck et al., (2018) to compute Y2H-SCORES and compare its performance to the reference methods. With regards to run time, they do not offer software nor raw datasets to execute and calculate run time. For Y2H-SCORES run time, most of the analysis took less than 10 minutes. The longest took up to 30 minutes. We configured Y2H-SCORES so run time will scale linearly with the number of baits that are analyzed. We achieved this by analyzing the baits in sets of 10, and then avoiding the exponential run time of analyzing pairwise bait contrasts that are required for calculating the specificity score. We explain this functionality in the software manual in GitHub https://github.com/Wiselab2/Y2H-SCORES/tree/master/Software.

- Have you ensured that when you randomize the data your approach cannot detect meaningful signal? Also, how robust is your approach to noise in the data (when you only randomize x% of the data, x=5%, 10%, 15%, ..., how much noise can your approach tolerate without dropping accuracy, at least without dropping it drastically)?

Authors response: This is a good point, S2 text explains these choices in the simulation. The ranges that we selected during the simulations are intended to resemble Y2H-NGIS datasets. All the data is randomized for every simulation, the percentages that we report are ranges for some parameters that define the interactors. For example, the strength of true interactions will be reflected in the relative fitness of the interacting yeast to the rest of the population (lines 262-268). Another example is the stickiness parameter, which noises the data by increasing autoactive preys in the library. The ranges for this parameter cover beyond what is been reported in previous studies (262-268, 314-325, S2 Text).

- The AUROC scores are close to 1 or 1, i.e., perfect. This is oddly suspicious. If this is correct, how to the authors explain this, especially given that AUPR scores aren't perfect? Also, how do the authors explain that in Fig. 2D, the three individual scores capture "different information" but each individual score yields perfect AUROC performance? If each score is perfect, then the three scores are identical and should capture the same information, no?

Authors response: The values in the ROC AUC curves are more stable because Y2H-NGIS validation sets are imbalanced (Saito 2015). We added this to the text as an explanation for the high performance at the ROC level (lines 303-304). PR AUC can be more informative depending on the situation, for example if the number of true positives is smaller than the number of true negatives, as happens in most Y2H-NGIS datasets. That is why we use both to describe the performance of the methods. We highlight the importance of using both metrics to report the performance in the benchmarking section, where we have cases higher ROC or PR curves depending on the dataset, as it is shown in Fig 5.

- Why was there a focus on a specific interaction for experimental validation? How was this particular interaction chosen? Did the authors test multiple interactions but only this one was validated, or did they just test this one? If this one was the only one that was tested, and it was tested because it was the highest-scoring predicted interaction, the authors should also test the lowest-scoring interaction to make sure that they cannot experimentally validate. Otherwise, without proper negative controls, the experimental validation of the interaction in question is a cherry-picked example that does not necessarily validate the proposed methodology.

Authors response: Thank you for this comment. For this resubmission, we removed the initial interaction and included a much more complete dataset using the MLA6 protein. We disclose all of the validation tests that we performed and all the positive and negative interactions that we found with the MLA6 baits. This contains 17 validated interactions and 38 negative tests with a wide range of scores. The results from this dataset allows the user to set a cut-off for the parameters of Y2H-SCORES that provides high confidence of a positive interaction, which was the original motivation for developing the software. Results from this section can be found at "Y2H-SCORES discards autoactive preys and identifies novel interactors of the barley MLA6 powdery mildew resistance protein". We are confident that the new data contained in the resubmission will satisfy the reviewers concern and more clearly demonstrate the applicability of our method to confidently predict true interactions form Y2H-NGIS data.

When the authors say "R code and ReadMe file for the Y2H-SCORES software" are provided, what exactly do they mean, i.e., what do they provide the code for? Which part of the study (what is the input, output, and goal of the code)? All code and data needed to be provided that allow the entire study (each and every step of the methodology and evaluation) to be fully reproduced. Is this already the case? A brief description in the paper what the provided code covers is needed.

Authors response: Initially we had the code for the Y2H-SCORES software alone. In this revision, we added code for the Y2H-Simulator and benchmarking with other Y2H-NGIS datasets. We added links to these in the methods.

R code and ReadMe file for the Y2H-SCORES software are provided at GitHub (https://github.com/Wiselab2/Y2H-SCORES/tree/master/Software). Users can find the instructions in the same repository. Y2H-NGIS code supporting the conclusions of this article are available at https://github.com/Wiselab2/Y2H-SCORES/tree/master/Publication.

Also, regarding reproducibility, descriptions in the text, including Methods, are often very vague (e.g., there is a brief statement which method is used and a reference is provided, but it's unclear exactly how that method was used, e.g., which parameter values were used). Much more detailed description of each methodological and evaluation step is needed for full study reproducibility.

Authors response: S2 Text contains all details about simulation methods and parameter choices, with a graphical summary in S5 Fig. We also added the experimental protocol in S3 Text with all the details of our Y2H-NGIS assays. Other details were added to the methods (lines 856-872, 890-896, 908-910, 913, 922-924). Each of the references in the methods contain full protocols of the methods followed by us.

Have all data underlying the figures and results presented in the manuscript been provided?
Large-scale datasets should be made available via a public repository as described in the *PLOS Computational Biology* data availability policy, and numerical data that underlies graphs or summary statistics should be provided in spreadsheet form as supporting information.

Reviewer #1: Yes

Reviewer #2: **No:** 1) Raw reads data (fastq files) of the Y2H-NGIS described in this study are not provided. These should be made available via a public repository such as SRA or ENA archives;

Authors response: Y2H-NGIS data supporting the conclusions of this article are available in NCBI's Gene Expression Omnibus (GEO) under the accession numbers GSE164814 (MLA61-161), GSE164815 (MLA61-225), GSE164816 (MLA6550-956), GSE164761 (luciferase - construct #1), GSE164762 (luciferase - construct #2).

We had uploaded the data previously and checked the box stating it would be released upon acceptance of publication, but apparently the reviewer was not made aware of this.

2) Data and information related to the different baits use to benchmark normalization methods and the Y2H-score algorithm are not made available which make difficult any reproducible research;

Authors response: Now we have made all the counts available in S1 Table.

3) Raw results output are not provided in supplementary files. Moreover the top candidates described in the paper are not made available.

Authors response: Ranking tables with the candidates are now available and provided in S8 Table.

Reviewer #3: Yes
